# CD44v3 is a marker of invasive cancer stem cells driving metastasis in gastric carcinoma

**DOI:** 10.1007/s10120-022-01357-y

**Published:** 2022-12-18

**Authors:** Julie Giraud, Lornella Seeneevassen, Benoit Rousseau, Damien Bouriez, Elodie Sifré, Alban Giese, Tra Ly Nguyen, Camille Tiffon, Yannick Lippi, Lamia Azzi-Martin, Julie Pannequin, Armelle Ménard, Emilie Bessède, Cathy Staedel, Francis Mégraud, Geneviève Belleannée, Philippe Lehours, Caroline Gronnier, Pierre Dubus, Christine Varon

**Affiliations:** 1 INSERM U1312, Bordeaux Institute of Oncology, University of Bordeaux, 146 rue Leo Saignat, 33076 Bordeaux, France; 2 Animal Facility, University of Bordeaux, 33076 Bordeaux, France; 3 Department of Digestive Surgery, Haut-Lévêque Hospital, 33000 Bordeaux, France; 4 CHU Bordeaux, 33076 Bordeaux, France; 5 Toxalim Research Centre in Food Toxicology, Université de Toulouse, INRAE, ENVT, INP-Purpan, UPS, Toulouse, France; 6 IGF, University of Montpellier, CNRS, INSERM, Montpellier, France; 7 INSERM U1212, ARNA, University of Bordeaux, 33076 Bordeaux, France; 8 Centre National de Référence des Campylobacters et Helicobacters, Pellegrin Hospital, 33076 Bordeaux, France; 9 Department of Histology and Pathology, Haut-Lévêque Hospital, 33000 Bordeaux, France

**Keywords:** Metastasis initiating cells, CD44 variant, Epithelial-mesenchymal transition, Gastric cancer, Cancer stem cells

## Abstract

**Background:**

Cancer stem cells (CSCs) are at the origin of tumour initiation and progression in gastric adenocarcinoma (GC). However, markers of metastasis-initiating cells remain unidentified in GC. In this study, we characterized CD44 variants expressed in GC and evaluated the tumorigenic and metastatic properties of CD44v3+ cells and their clinical significance in GC patients.

**Methods:**

Using GC cell lines and patient-derived xenografts, we evaluated CD44+ and CD44v3+ GC cells molecular signature and their tumorigenic, chemoresistance, invasive and metastatic properties, and expression in patients-derived tissues.

**Results:**

CD44v3+ cells, which represented a subpopulation of CD44+ cells, were detected in advanced preneoplastic lesions and presented CSCs chemoresistance and tumorigenic properties in vitro and in vivo. Molecular and functional analyses revealed two subpopulations of gastric CSCs: CD44v3+ CSCs with an epithelial-mesenchymal transition (EMT)-like signature, and CD44+/v3– CSCs with an epithelial-like signature; both were tumorigenic but CD44v3+ cells showed higher invasive and metastatic properties in vivo. CD44v3+ cells detected in the primary tumours of GC patients were associated with a worse prognosis.

**Conclusion:**

CD44v3 is a marker of a subpopulation of CSCs with metastatic properties in GC. The identification of metastasis-initiating cells in GC represents a major advance for further development of anti-metastatic therapeutic strategies.

**Supplementary Information:**

The online version contains supplementary material available at 10.1007/s10120-022-01357-y.

## Introduction

Gastric cancer, mainly composed of gastric adenocarcinoma (GC) represents the fourth leading cause of cancer deaths worldwide [1]. It is frequently detected too late, at advanced metastatic stages. Current treatment is based on surgery with conventional chemo and radio-therapies, but for unresectable metastatic GC cases, therapeutic options are usually limited to palliative chemotherapy with a 5 years survival rate of less than 4%. GC displays molecular heterogeneity, driven by genetic and epigenetic modifications [2], which likely gives rise to subpopulation of cells with different tumorigenic and chemoresistance properties. At the top of cellular hierarchy within tumours, the so-called cancer stem cells (CSC) give rise via asymmetrical division and differentiation properties to the more or less differentiated cells composing the heterogeneous tumour mass. CSCs biomarkers are diverse and depend on the organs of origin of the tumours. Nevertheless, CD44 transmembrane glycoproteins are one of the most relevant CSCs markers in solid cancers [3] including GC [4–6]. However, antibodies used to detect CD44 in these studies recognize epitopes located in constant regions of CD44 (panCD44) and cannot discriminate CD44 isoforms (CD44v). Human CD44 proteins are encoded by a single gene containing 19 exons, among which 12 exons can be alternatively spliced to give rise to hundreds of CD44v proteins [7]. CD44 receptor displays multiple roles in cell–cell and cell-extracellular matrix adhesion, proliferation and migration. CD44v have been described to act as co-receptors for growth factors and as platforms for matrix metalloproteinase activity and growth factor precursor cleavage [8]. CD44 exon v3 contains a unique and conserved [9] heparan sulfate (HS) consensus motif, located in the extracellular domain of the receptor, for attachment to HS proteoglycans. This domain can bind to HS-dependent growth factors, such as hepatocyte growth factor, heparin-binding epidermal growth factor-like, basic Fibroblast Growth Factor and Vascular Endothelial Growth Factor [10–12] thereby increasing their local concentration for signal transduction. These properties are critical for CD44v3-containing isoforms’ capacity to control cell survival, migration, tumour progression and metastatic dissemination [13–15]. The expression of panCD44 correlates with poor prognosis in patients with intestinal type GC [16, 17]. Lau et al*.* have shown that CD44v8-10 was the predominant CD44 variant expressed in GC cells and can be a gastric CSCs marker [17]. However, panCD44 or CD44v8-10 are simply markers allowing the enrichment of gastric CSCs since only a fraction of these cells can form tumorspheres in vitro and tumours in vivo in xenograft models [4, 6, 17], highlighting the need to identify more specific CSCs markers. CD44v3 is much less expressed than panCD44 and CD44v8-10 in adult tissues and is upregulated in human colon tumours and metastases [15]. CD44v3 is expressed by CSCs in human oral squamous cell carcinoma [18] and head and neck squamous cell carcinomas (HNSCC) [19]. Nonetheless, the clinical significance of CD44v3 expression in GC was reported only in few studies with controversial results [20–22], and no information is available regarding its functional role in gastric CSCs.

In this study, we aimed to decipher the cellular heterogeneity of GC cells in terms of CD44 variants expression, and especially to study the properties of cells expressing CD44v3.

## Methods

We declare that all authors had access to the study data and have reviewed and approved the final manuscript.

### Ethic statements on human samples and animal experiments

Animal experiments with 6 week-old female NSG immunodeficient mice were performed in level 2 animal facility of the University of Bordeaux (accreditation number B33-063-916 received on 23/05/2016) and were approved by the local Ethic Committee on Animal Experiments CEEA50 of Bordeaux (reference A12005/2017103118319700v7). Paraffin-embedded tissue samples from 179 GC patients were obtained in agreement with the Direction for Clinical Research and the Tumour and Cell Bank of the University Hospital Centre of Bordeaux (Haut-Leveque Hospital), as previously reported [4, 23]. Paired tumour and non-tumour tissue samples were obtained from stomach surgical resection in GC patients; non-tumour gastric mucosa were taken distant from the tumour site (limits of exeresis) and were healthy or involving different pre-neoplastic lesions (chronic atrophic gastritis, intestinal metaplasia, dysplasia, scored according to the Sydney System criteria).

### Patient and public involvement

It was not appropriate to involve patients or the public in the plans of our research.

### Histology and immunohistochemistry staining

Primary antibodies used were as follows: anti-human CD44 1:100 (BD Pharmingen 550392), anti-human CD44v3 1:3000 (R&D, clone 3G5), anti-human CD44v6 1:500 (R&D, clone 2F10), anti-human ALDH1 1:400 (BD 611194, clone G44) and anti-Ki67 1:75 (Agilent M7240). CD44 [23] and CD44v3 expressions were scored in percentage of positive cells (mostly at the plasma membrane and more rarely in the cytoplasm and nucleus) determined in a double blinded lecture: score 0, no expression, score 1, 1–5%, score 2, 5–20%, score 3, 20–50%, score 4 > 50% of positive cells. CD44 and CD44v3 expression intensities were also scored as follows: 1, lowest; 2, median and 3, highest stain intensities.

**Table 1:** Gastric CSC frequencies determined on the capacity of FACS-sorted cells to develop a tumour after xenograft in limiting dilutions in NSG mice

Cases		Marker	Number of tumors/number of transplanted mice								Gastric CSC frequencies (95% confidence interval)		Test for differences in CSC frequencies compared to EpCAM+ CD44v3
			Number of transplanted cells										
			5000	1500	1000	500	300	150	100	30			
GC04	EpCAM+	panCD44-	3/5	0/5		0/4		0/5			1/9021	(1/27281–1/2983)	*P* = 0.00338
	EpCAM+	panCD44+ /v3-	5/5	5/5		4/4		4/5			1/91	(1/251–1/33)	*P* = 2.06e-05
	EpCAM+	CD44v3+	5/5	3/5		2/5		0/5			1/1378	(1/2918–1/651)	n.a
GC10	EpCAM+	panCD44-			2/5		0/5		0/5	0/5	1/3174	(1/11922–1/779)	*P* = 0.0124
	EpCAM+	panCD44+ /v3-			5/5		5/5		4/5	3/5	1/48	(1/108–1/21)	*P* = 1.56e-05
	EpCAM+	CD44v3+			4/5		2/5		2/5	0/5	1/514	(1/1109–1/238)	n.a

*n.a* not applicable

### Gastric epithelial cell lines culture and patient-derived tumour xenografts

GC04, GC06, GC07, GC10, GC35 and GC44 are patient-derived GC, which were successfully established by serial subcutaneous transplantation of tumour pieces in NSG immunodeficient mice as previously described [4]. GC04, GC07, GC10, GC35 and GC44 are intestinal type GC, while GC06 is a diffuse-type GC, as previously reported [4].

### Flow cytometry and fluorescence-activated cell sorting

100,000 cells were stained with Aldefluor^®^ reagent and then with anti-human CD44-PE as previously reported [4, 23] and CD44v3-APC. Flow cytometry was performed using BD FACSCanto II or Fortessa instruments and DIVA analysis software (BD). FACS-sorted cells were performed after dissociation of PDX tumours with human tumour dissociation enzyme and GentleMACS dissociator (all from Miltenyi). 7-amino actinomycin (BD) positive dead cells were excluded and EpCAM^+^panCD44^+^/CD44v3^+^ and EpCAM^+^panCD44^−^/CD44v3^−^ were sorted using FACS Aria II instrument. Antibodies used were 1:100 EpCAM-Vioblue (Miltenyi), 1:20 CD44-APC or CD44-PE (BD clone G44-26) or CD44v9-PE (Biolegend), 1:15 CD44v3-APC (R&D clone 3G5) and 1:50 CD44v6-PE vio770 (Miltenyi).

### Microarray gene expression studies and statistical data analysis

Detailed information is provided in Supplemental Methods.

### CD44 exon-specific PCR

CD44 exon-specific PCRs were performed as previously reported [24].

### siRNA transfection

Cells were grown in 6-well plates and transfection of 25 nmol/L siRNA was performed using Lipofectamine RNAiMAX (Thermo Fisher Scientific) according to manufacturer’s instructions [23]. Two rounds of siRNA transfections were performed. siRNA sequences are in Supplementary Table 3.

### Tumorsphere culture

250–1000 cells (as indicated in figure legends) were seeded in non-adherent 96-well culture plates, as previously described [4, 23], in serum-free GlutaMAX-DMEM/F12 medium supplemented with 20 ng/mL of epidermal growth factor, 20 ng/mL of basic- fibroblast growth factor, 0.3% glucose, 5 μg/mL of insulin, 1:100 N2 supplement (all from Invitrogen and Sigma) and cultured at 37 °C in a humidified 5% CO2 atmosphere.

### In vivo xenograft experiments and extreme limiting dilution assay

GC04 and GC10 PDX tumours were dissociated and EpCAM^+^/panCD44^+^/CD44v3^−^, EpCAM^+^/ panCD44^+^/CD44v3^+^ and EpCAM^+^/panCD44^−/^CD44v3^−^ cells freshly sorted by FACS were suspended in 100 μL of 7 mg/ mL ice-cold Matrigel (Ozyme) and subcutaneously injected into the dorsal flank of 6-weeks-old NSG female mice. Increasing number of cells (30; 100; 300 or 1000 cells for GC10 and 50; 500; 1500 and 5000 cells for GC04) was injected in 5 mice per condition. Tumours were monitored twice a week up to 12 weeks using a calliper. Tumours were collected, fixed in a 3.7% buffered-formaldehyde solution and embedded in paraffin following standard procedures as described [4, 23, 25].

### Orthotopic xenograft of FACS-sorted cells

GC10 PDX cells and MKN45 cells, previously transduced with a lentivirus encoding luciferase gene [25], were grown subcutaneously in NSG mice and tumours were dissociated and immuno-stained for FACS cell sorting as described in upper sections. 10000 GC10 cells and 2500 CD44v3+ and CD44v3– MKN45 cells were injected into the sub-serosa of the stomach of 6 weeks old male NSG mice [25]. Surgery procedures are detailed in Supplemental Methods.

Detailed methods about gastric epithelial cell lines culture, invasion and gelatine degradation assay, histological procedures, RNA extraction, RT-qPCR analysis and statistical analysis are provided in Supplemental Methods.

## Results

### Characterization of CD44 variants in GC

The expression of CD44 isoforms was deciphered in GC cell lines and in-house patient-derived xenografts (PDX) [4] using exon-specific RT-PCR [24]. Interestingly, mRNAs encoding CD44v3-containing isoforms (CD44v3) were expressed in GC cell lines and PDX-derived cells (Supplementary FigureS1A). Exon v3 was present in combination with all other exons (CD44v2/3-10) and in direct association with exons v8 to v10 [named CD44v3,8-10 or CD44v3E, as described by Qiu et al. [26]]. Consistent with previous reports [20, 21], mRNA levels of global CD44 (CD44t) and of two common isoforms, the standard isoform CD44s (the shorter isoform, containing no variable exon) and the epithelial isoform CD44E (expressed in epithelia, containing the variable exons v8 to v10 and also named CD44v8-10), were detected, even at low levels, both in normal healthy mucosa of the stomach (fundus and antrum) and in GC cell lines and PDX-derived cells while CD44v3 expression was restricted to GC (Supplementary Fig.S1B).

### CD44v3+ cells correspond to a subpopulation of CD44+ cells that is rare in healthy gastric epithelium, but present in GC

**Fig. 1:**
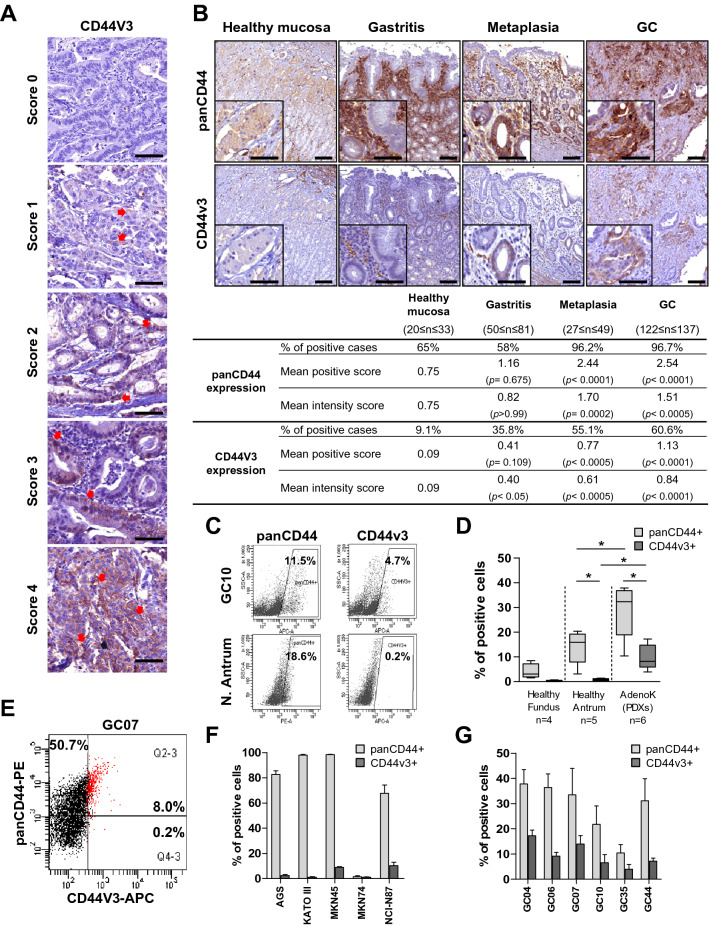
CD44v3+ cells are rare in healthy gastric epithelium but detected in GC. **A** Representative images of immunohistochemistry staining of panCD44 and CD44v3 in GC tissues according to the scores of percentage of positive cells. **B** Representative images and relative quantification of panCD44 and CD44v3 expression on healthy tissues, gastritis, metaplasia and GC (20 ≤ *n* ≤ 137 cases). Scale bars, 100 µm. Values represent percentages of positive cases and mean positive and intensity scores with P values (*P*) calculated versus healthy mucosa. **C** Representative flow cytometry profiles for panCD44 and CD44v3 on gated EpCAM+ cells of GC10 PDX and normal antrum. **D** Percentage of CD44v3+ and panCD44+ cells determined by flow cytometry on cells dissociated from gastrectomy tissues (healthy fundus and antrum) and PDX cells. Min to max from 4 ≤ *n* ≤ 6 independent experiments. **E–G** Representative profile of expression (**E**, GC07) and quantification of the percentage of CD44v3+ and panCD44+ cells determined by flow cytometry on adherent GC cell lines (**E–F**) and on gated EpCAM+ PDX cells isolated from fresh tumours (**G**). *n* = 3 independent experiments, mean ± S.E.M, except GC35 and GC44, *n* = 2

The expression of CD44v3 and panCD44 (corresponding to all CD44 isoforms) was evaluated in tissue micro arrays (TMA) composed of stage I-IV GC and paired non-tumoral gastric mucosa from 137 GC patients using immunohistochemistry (Fig. 1A–B). CD44v3+ cells were rare in the healthy gastric epithelium and detected in only 9.1% of cases compared to CD44, detected in 65% of cases (Fig. 1B). In preneoplastic lesions, CD44v3+ cases progressively increased to reach 55.1% in intestinal metaplasia and 60.6% in GC compared to CD44 which was detected in more than 96% of cases as from the stage of intestinal metaplasia. The same trend was observed for panCD44 and CD44v3 staining intensity demonstrating that the rare CD44v3+ cells present in the healthy tissue expressed the protein in lower amount, which increased with the disease progression. These results show that CD44v3 expression remained rare, and was detected later and in more advanced preneoplastic lesions than CD44, suggesting that it could be a more relevant biomarker of disease progression than CD44 (Fig. 1B).

The expression of CD44v3 and panCD44 was then evaluated in cells dissociated from gastric mucosa tissues, GC cell lines and PDX-derived cells by flow cytometry (Fig. 1C–G). The detection of Epithelial Cell Adhesion Molecule (EpCAM) was used to selectively analyse epithelial cells from gastric tissues. In healthy gastric mucosa, CD44v3+ epithelial cells were absent in fundus and rare in antral mucosa (< 1% of cells), whereas panCD44+ epithelial cells represented 4% of cells in fundus and 14% in antrum (Fig. 1C–D). In GC, CD44v3 was expressed > 3 times less than panCD44, in ≤ 17% of cells in the 5 GC cell lines and the 6 PDX analysed compared to panCD44 whose expression reached 98% of GC cells and 37% of PDX cells (Fig. 1E–G). Note that there was no difference in terms of CD44v3+ cells between diffuse-type GC (MKN45, AGS, Kato-III and GC06) and intestinal-type GC (MKN74, NCIN87, GC04, GC07, GC10, GC35 and GC44). Taken together, these results highlight the cellular heterogeneity inside the population of panCD44+ GC cells and show that, contrarily to panCD44, CD44v3+ cells correspond to a rare subpopulation of cells in GC that is primarily absent in healthy gastric mucosa.

### The subpopulation of CD44v3+ GC cells display CSC properties

**Fig. 2:**
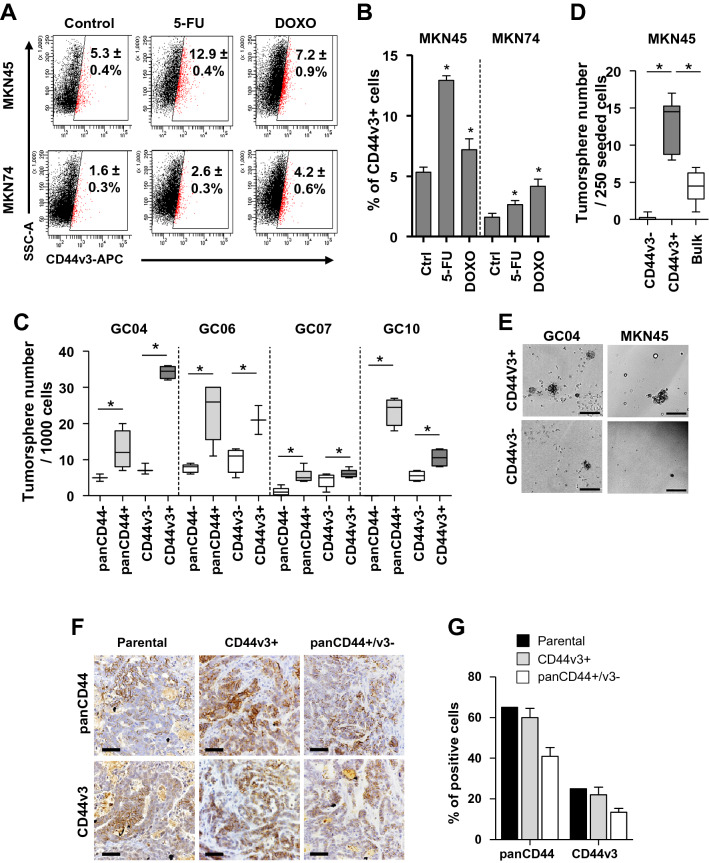
CD44v3+ cells from GC cell lines and PDX display CSC properties. **A–B** Representative flow cytometry profiles (**A**) and quantification (**B**) of CD44v3 (gated on live 7AAD- cells) on 48 h 5-fluorouracil (5-FU) and doxorubicin (DOXO)-treated or untreated (control) cells. Data are mean ± SEM of 3–4 independent experiments in triplicates. **C-D** Tumorsphere formation by (**C**) FACS-sorted EpCAM+/panCD44+, CD44v3+ , panCD44- and CD44v3- PDX cells and (**D**) FACS-sorted CD44v3+, CD44v3- and bulk MKN45 cells. Data are min to max from ≥ 3 independent experiments. **E** Representative images of tumorsphere formed. **F-G** Representative images of immunohistochemistry (**F**) and quantification (**G**) of CD44v3+ and panCD44+ cells in GC10 parental tumours and those initiated by CD44v3+ and panCD44+/CD44v3- FACS-sorted cells. **E–F** Scale bars, 100 µm

We previously reported that panCD44+ cells display all the hallmarks of CSCs since they have tumorigenic properties both in vitro (give rise to tumorspheres*)* and in vivo (give rise to tumours after xenograft in immunodeficient mice), and are chemo-resistant [4, 27, 28]. Here, we found that after 48 h treatment with 5-fluorouracil or doxorubicin, resistant GC cells highly expressed CD44v3 both at the mRNA (Supplemental Fig.S2) and protein (Fig. 2A–B) levels. These chemoresistance properties were not restricted only to CD44v3+ cells as CD44s and CD44E expression also increased following chemotherapy (Supplementary Fig.S2). To evaluate tumorigenic properties associated to CSCs in vitro, panCD44+ , panCD44-, CD44v3+ and CD44v3– cells were isolated by FACS and submitted to tumorsphere assay (Fig. 2C–E)*.* Both panCD44+ and CD44v3+ cells formed significantly more tumorspheres than their negative counterparts (Fig. 2C–E). The ability of CD44v3+ cells to form tumorsphere was either higher (in GC04), the same (in GC06 and GC07) or lower (in GC10) than panCD44+ cells (Fig. 2C), suggesting that CD44v3+ cells have similar tumorigenic properties than panCD44+ cells. To confirm this result in vivo, subcutaneous xenograft experiments in immunodeficient mice were performed with GC04 and GC10 PDX FACS-sorted EpCAM+ GC cells expressing or not panCD44 and CD44v3 using the extreme limiting dilution analysis (ELDA) method as previously reported [4] (Table 1). CSCs frequency in CD44v3+ cells was significantly higher than panCD44- cells, but lower than panCD44+ /CD44v3– cells (Table 1). Importantly, in both cases tumours were heterogeneous, composed of panCD44 and CD44v3 positive and negative cells (Fig. 2F–G), showing that both CD44v3+ and panCD44+/v3– cells have self-renewal and asymmetrical division and differentiation properties that are hallmarks of CSCs.

Altogether, these results show that both CD44v3+ and panCD44+ /v3– GC cells have CSCs tumorigenic and differentiation properties.

### CD44v3+ cells represent a subpopulation of panCD44+ cells with an epithelial-mesenchymal transition molecular signature

**Fig. 3:**
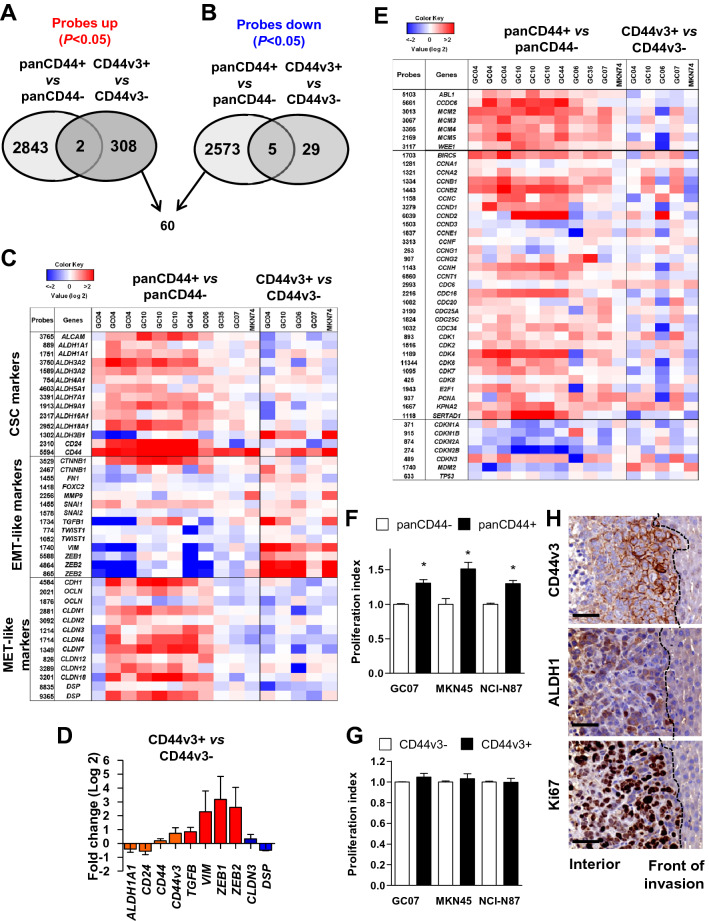
panCD44 and CD44v3 identify distinct CSCs with different EMT/MET expression profiles and proliferative states. **A-B** Venn diagram representing the number of probes upregulated (**A**) and downregulated (**B**) in panCD44+ *versus* panCD44- cells and CD44v3+ versus CD44v3- cells (*P* < 0.05). **C** Heatmap of CSC and EMT/MET genes (n = 6 PDXs for panCD44 and n = 4 PDXs for CD44v3, with MKN74). **D** Relative mRNA expression determined by qRT-PCR in CD44v3+ *versus* CD44v3- MKN45 cells. (*n* = 3 independent experiments, mean ± S.E.M). **E** Heatmap of cell cycle gene signature as described in (**C**). Data correspond to most expressed probes for each gene. **F-G** Proliferation index determined by flow cytometry after 48 h culture using CellTrace™ in panCD44+ (**F**) and CD44v3+ (**G**) cells and their respective negative counterparts. (*n* = 3 independent experiments, mean ± S.E.M.). **H** Representative immunohistochemistry images of human CD44v3, ALDH1 and Ki67 stainings (brown) in MKN45 liver metastases in orthotopically xenografted mice. Scale bars, 50 µm

Transcriptomic analysis was conducted on FACS-sorted subpopulations of GC cells expressing or not panCD44 or CD44v3 from 4 to 6 established PDX and MKN74 GC cell line using microarrays. Unsupervised hierarchical clustering of differentially expressed genes between positive and negative cells strongly separated samples in 2 groups based on their panCD44 and CD44v3 origin (Supplementary Fig.S3). PanCD44+ and CD44v3+ subpopulations of GC cells expressed distinct patterns of genes (detailed in supplementary materials and Supplementary Table S4–6). Among the 310 mRNA transcripts that were significantly upregulated in CD44v3+, 60 were significantly downregulated in panCD44+ (Fig. 3A–B, Supplementary Table S6). Interestingly, many of them are involved in EMT, invasion and tumour aggressiveness. Liu et al. have reported that breast CSCs exist in two distinct states: one state composed of invasive EMT-like CSCs characterized as CD24-CD44+, and one state corresponding to proliferative and more epithelial MET-like CSCs expressing ALDH activity [29]. These EMT-like and MET-like signatures [29] were analysed in our GC transcriptomic analyses (Fig. 3C). In GC, we previously reported that gastric CSC express CD44, CD166 (ALCAM), CD24, and ALDH activity [4, 28] which was confirmed here in panCD44+ versus panCD44- cells but not in CD44v3+ versus CD44v3– cells. EMT-like- associated transcripts such as those coding for TGF-β1, Vimentin, ZEB1 and ZEB2 were enriched in CD44v3+ cells and downregulated in panCD44+ cells. Conversely, MET-like transcripts including those encoding E-cadherin (*CDH1*), Occludin (*OCLN*), several Claudins (*CLDN*) and Desmoplakin (*DSP*) were increased in panCD44+ cells and diminished in CD44v3+ cells (Fig. 3C). Variations in the expression of a subset of those genes were confirmed by qRT-PCR in MKN45 FACS-sorted CD44v3+ cells compared to CD44v3– cells (Fig. 3D).

The proliferative state of these subpopulations was evaluated. PanCD44+ cells harboured a positive cell cycle progression molecular signature compared to panCD44- cells (upregulation of genes regulating S phase entry, DNA replication and G2/M transition, with downregulation of cyclin-dependent kinases inhibitors), but not CD44v3+ versus CD44v3– cells (Fig. 3E). Flow cytometry analyses of cell proliferation confirmed that panCD44+ cells were more proliferative than panCD44- cells (Fig. 3F) whereas CD44v3+ cells were as proliferative as CD44v3– cells (Fig. 3G). Interestingly, in tissues, CD44v3+ GC cells were localized at the invasion front of GC liver metastases, whereas ALDH1+ GC cells were more centrally located within the tumour mass as described by Liu et al.in breast cancer for the MET-like CSCs [29], but both expressed Ki67 proliferation marker (Fig. 3H).

**Fig. 4:**
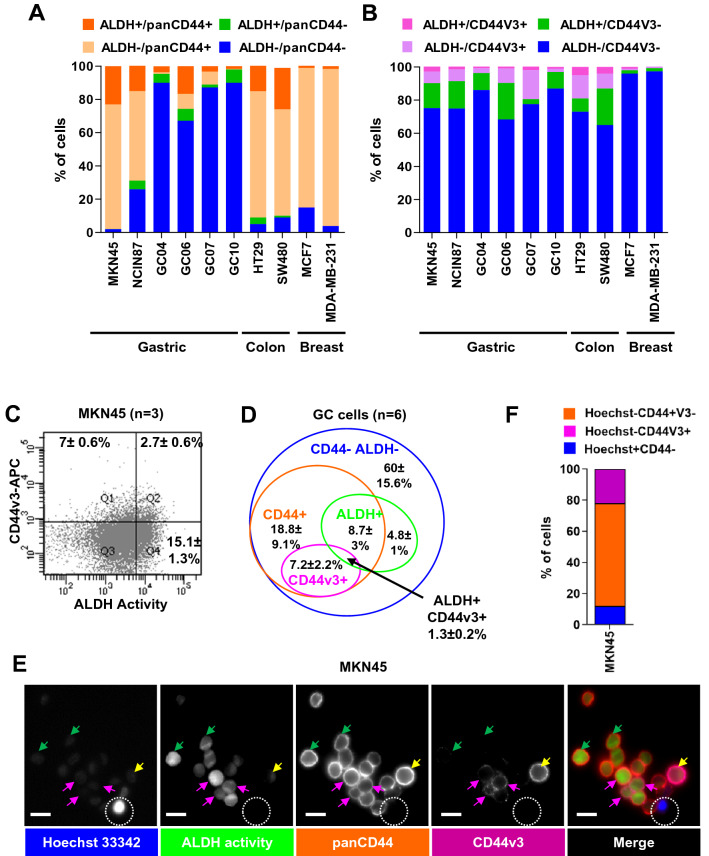
ALDH+ cells are mainly panCD44+ and CD44v3-. **A-B** Cumulated percentages of cells according to panCD44 expression and ALDH activity (**A**) and to CD44v3 expression and ALDH activity (**B**) in gastric, colon and breast cancer cell lines. All cell lines were cultivated in conventional adherent culture conditions, and PDX-derived GC cells (GC04, GC06, GC07, GC10) were collected from tumor xenografts for direct flow cytometry analysis. Data are mean of 2–3 independent experiments. **C** Representative flow cytometry profiles for CD44v3 expression and ALDH activity on MKN45 cells (*n* = 3 independent experiments, mean ± SEM). **D** The diagram represents the mean percentages of each cell subpopulations and the overlap between the ALDEFLUOR+ phenotype (ALDH+) and the panCD44+ and CD44v3+ phenotypes of GC cells related to (**A** and **B)** (*n* = 6 GC cases, mean ± SEM). **E** Representative images of CD44-PE staining (panCD44, dark orange), CD44v3-APC (purple), ALDH activity detected by ALDEFLUOR reagent (green) and nuclei staining with Hoechst-33342 (blue) on live 5 days MKN45 tumorspheres. White dotted circle points out a cell positive for Hoechst-33342 nuclear staining but negative for ALDH, CD44 and CD44v3. Pink arrows, Hoechst-/ALDH+/panCD44+/CD44v3+ cells. Green arrows, Hoechst-/ALDH+/panCD44+/CD44v3- cells. Yellow arrows, ALDH-/panCD44+/CD44v3+ cells. Bars, 10 µm. **F** Quantification of cells related to experiment shown in (**E**). Note the absence of Hoechst+CD44+ cells. *n* = 81 cells analysed inside tumorspheres

Flow cytometry experiments confirmed that ALDH+ GC cells were mainly panCD44+ (dark orange bars compared to green bars, Fig. 4A) and CD44v3– (dark pink bars versus green bars, Fig. 4B–C). CD44v3+ cells were mainly ALDH- (light pink versus dark pink bars), and CD44v3+/ALDH+ cells (dark pink bars) accounted only for 1.3 ± 0.2% of GC cells (Fig. 4B–D). We observed the same tendency for breast and colon cancer cell lines, in which a rare subpopulation of CD44v3+/ALDH+ was detected (1–5%, respectively, Fig. 4A–B). These results were confirmed on live MKN45 tumorspheres, in which CD44v3+ cells were either ALDH+ (pink arrows) or ALDH- (yellow arrows). The Hoechst 33342- cells corresponded to CSCs with drug efflux properties whereas Hoechst 33342+ cells corresponded to more differentiated non-CSC cells as described previously [4, 28] (Fig. 4E–F). These Hoechst 33342– cells were either CD44v3+ or panCD44+/v3– (Fig. 4F), suggesting that both CD44v3+ and panCD44v3– cells have drug efflux properties. This was confirmed in chemoresistance assays, in which both CD44v3+ and panCD44+/v3– FACS-sorted MKN45 cells presented similar resistance to treatment in response to 5-fluorouracil and doxorubicin (Supplemental Fig.S4B).

Taken together, these results suggest that the subpopulation of GC cells expressing CD44v3 may correspond to EMT-like CSCs located at the invasive front of tumours while panCD44+ cells may correspond to more proliferative MET-like CSCs within the tumour mass.

### CD44v3+ subpopulation of gastric CSC drives metastasis

**Fig. 5:**
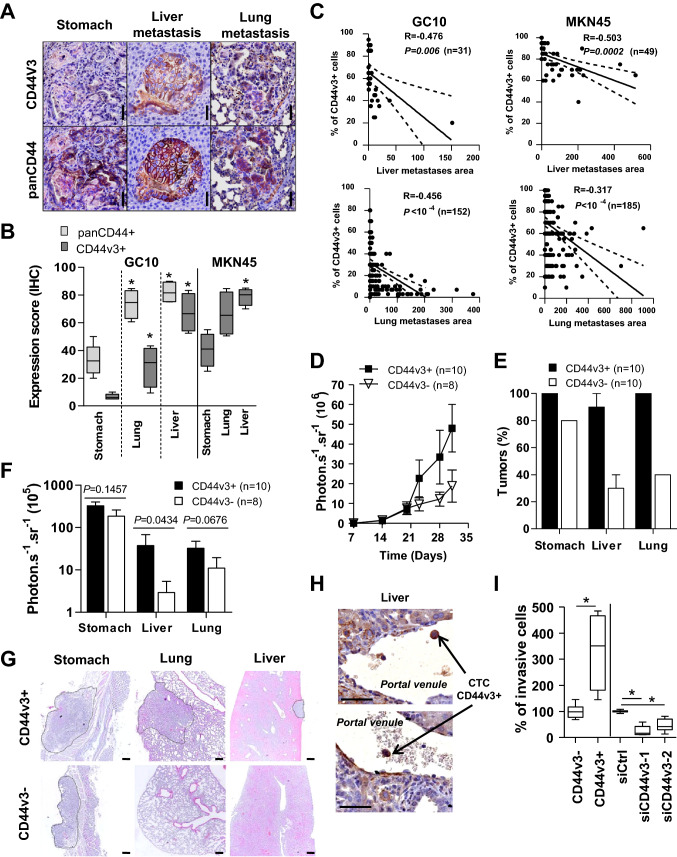
CD44v3+ cells drives GC metastasis. **A** Representative images of panCD44 and CD44v3 immunohistochemistry staining on stomach tumours and metastases 8 weeks after GC10 orthotopic xenograft. Scale bars, 50 µm. **B** Quantification of panCD44+ and CD44v3+ cells in stomach tumours and metastases obtained 8 (GC10) and 4 (MKN45) weeks after orthotopic xenograft. (**C**) Correlation of metastases areas (× 10^4^ µm^2^) with percentage of CD44v3+ cells according to (**A**) and (**B**). *n* = 4 mice. **D** Kinetic of in vivo whole-body bioluminescence imaging analysis of orthotopic tumours and metastases growth after xenograft of 2500 CD44v3+ and CD44v3- FACS-sorted MKN45-luciferase expressing cells. Data are mean ± SEM of 2 independent experiments. *n* = 10 mice per group (2 mice xenografted with CD44v3- cells did not develop stomach tumours). **E** Percentage of mice developing primary tumours and metastases 4.5 weeks after orthotopic xenograft, and **F** analysis of bioluminescence imaging. **G** H&E staining of tumours (dotted circles) from (**E**). Scale bars, 200 µm. (**H**) Representative immunohistochemistry images of CD44v3+ cells in portal venules of orthotopically xenografted mice with MKN45 cells. Scale bars, 50 µm. **I** Percentage of invasive cells from MKN45 CD44v3- and CD44v3+ FACS-sorted and bulk cells transfected with siRNA against CD44v3 (siCD44v3-1, siCD44v3-2) or scrambled siRNA (siCtrl), counted 18 h after cell seeding on inserts. Data are min to max of ≥ 2 independent experiments in duplicates

Since CD44v3+ GC cells harbours an EMT-like signature and CD44v3–containing isoforms have been involved in metastatic progression in breast cancer [30, 31], we next evaluated their invasive and metastatic properties in GC. First, CD44v3 expression was evaluated by immunohistochemistry in orthotopic xenograft mouse models of GC developing distant metastases [25]. An enrichment of CD44v3+ cells in liver and lung metastases was observed compared to primary gastric tumours (Fig. 5A–B). CD44v3 was more strongly expressed in micro-metastases (Fig. 5A) than in bigger metastases, and the percentage of CD44v3+ cells was inversely correlated with the size of lung and liver metastases (Fig. 5C). Similar results were obtained for panCD44+ cells (Fig. 5A–B, Supplemental Fig.S4B). Secondly, orthotopic xenograft experiments were reproduced with MKN45 FACS-sorted CD44v3+ and CD44v3– cells. CD44v3+ cells were more tumorigenic than CD44v3– cells (Fig. 5D) and generated both stomach tumours (10/10) and distant metastases (liver, 9/10; lung, 10/10) (Fig. 5E), whereas CD44v3– cells grew locally in the stomach (8/10) and disseminated at a lower frequency (liver, 3/10; lung, 4/10) (Fig. 5E). Both quantitative bioluminescence imaging and histological analysis (Fig. 5F–G) showed that liver and lung metastases derived from CD44v3+ cells were bigger than those derived from CD44v3– cells while stomach tumours were similar.

In addition, CD44v3+ cells were detected at the invasive front of macro-metastases (Fig. 3H). Accordingly, circulating tumour cells (CTC) detected in portal venules of mice developing liver metastases expressed CD44v3 (Fig. 5H). Furthermore, this correlated with in vitro observations where CD44v3+ cells isolated by FACS were more invasive (Fig. 5I) and CD44v3 expression inhibition using RNA interference inhibited their invasive properties in collagen-coated Boyden’s chamber (Fig. 5I, Supplemental Fig.S5A–C) as well as gelatine degradation properties (Supplemental Fig.S5D).

Altogether, these results suggest that GC metastatic dissemination and colonization of distant organs may originate mainly from CD44v3+ cells.

### CD44v3 expression in GC cells is correlated with metastasis in GC patients and a poor overall survival

**Fig. 6:**
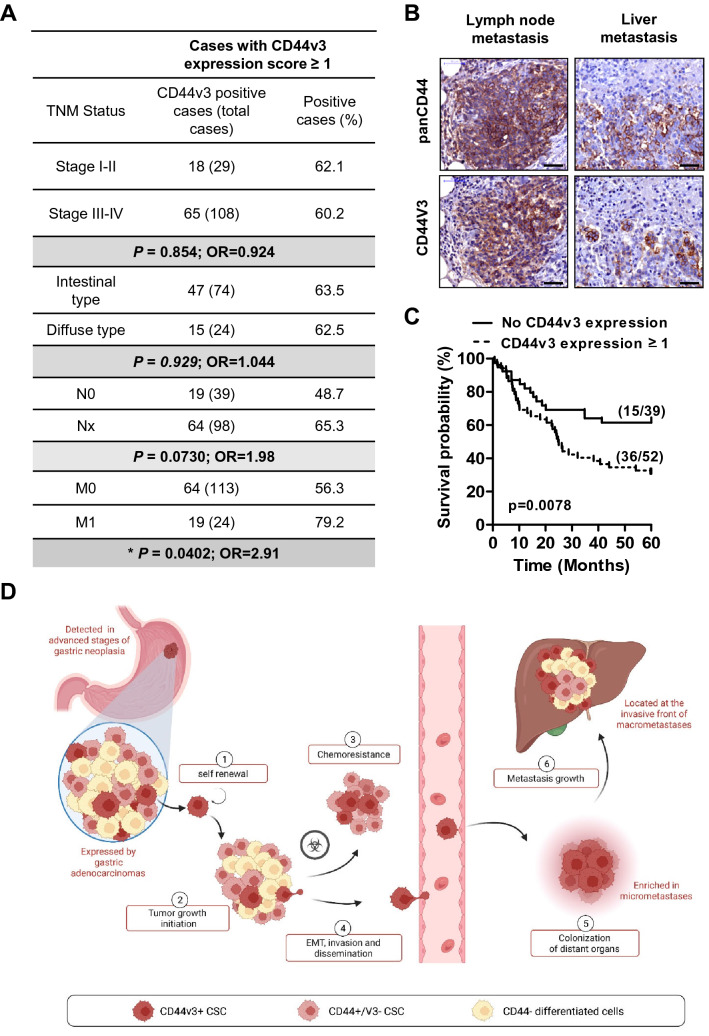
CD44v3 expression is associated with invasive stage and poor 5 years survival rate in GC patients. **A** Association tests of CD44v3 expression (score ≥ 1) with the TNM stage of GC patients (*n* = 137) and Lauren histological classification (*n* = 98). T, tumour stage I to IV; N0, no lymph node invasion, N1, lymph node invasion; M0, no metastasis, M1, metastasis. OR, Odds Ratio, Pearson’s Chi square tests. **B** Representative images of immunohistochemistry staining of panCD44 and CD44v3 on disseminated tumour cells in lymph node and liver in GC patient. Scale bars, 50 µm. **C** Kaplan–Meier survival curves according to CD44v3 expression (score ≥ 1). *n* = 91 patients for whom survival data were available. **D** Graphical representation of CD44v3+ cancer cells properties in GC progression and dissemination

The clinical relevance of CD44v3 expression as a putative biomarker of invasiveness and prognosis was explored by immunohistochemistry on a local collection of GC patients tumour tissues using TMA (*n* = 137 cases) (Fig. 6). CD44v3+ expression in GC was neither associated with tumour stage nor Lauren histological classification nor with lymph node invasion, but was significantly associated with the metastasis status, CD44v3 expression being detected in 79.2% of metastatic patients compared to 56.3% in non-metastatic patients (OR = 2.91, *P* = 0.0402) (Fig. 6A). CD44v3+ GC cells were detected in lymph nodes and liver metastases of GC patients, and CD44v3 expression in GC tumours was significantly associated with a poor 5 years overall survival (*P* = 0.0078, *n* = 91) (Fig. 6B–C).

A previous study in colon cancer demonstrated that CD44v6 is a marker of CSC-driving metastasis and that its expression is correlated with poorer prognosis [32, 33]. Interestingly, in GC cell lines and PDX-derived cells, CD44v3+ cells represented a subpopulation among CD44v6+ v9+ cells (Supplemental Fig.S6A). Combined with CD44v-exon-specific PCR analysis (Supplemental Fig.S1A), our data show that CD44v3+ cells are different from CD44E+ cells, and express the long isoforms CD44v3-10 and potentially CD44v3E which may be co-expressed in the same cells*.* This was confirmed on PDX tumour tissue sections in which CD44v3 detection marked a sub-population of CD44v6+ cells (Supplemental Fig.S6). Similar results were observed for colon and breast cancer cell lines with the presence of a rare subpopulation of CD44v3+/v6+ cells, representing less than 10% of CD44v6+ cells (Supplemental Fig.S6) and detected both in primary tumours and metastases of colon and breast cancer patients (Supplemental Fig.S7).

Taken together these results suggest that CD44v3+ cells represent a subpopulation of panCD44+ and CD44v6+ v9+ cancer cells in gastric, breast and colorectal cancers, and that the detection of CD44v3+ GC cells could be a marker of aggressiveness and poor prognosis in GC.

## Discussion

In this study, we show that different CD44 isoforms are expressed in GC cells lines and PDX, including the standard isoform CD44s, the epithelial isoform CD44E and CD44v3 isoforms. CD44E has been previously described as a CSC marker in GC; however, as ~ 80% of panCD44+ cells express CD44E isoform in GC [17] but only ~ 3,5% are CSCs [4], CD44E expression is not restricted to CSCs. Here we show that CD44v3 is not expressed in healthy gastric mucosa and appears progressively in pre-neoplastic lesions, reaching 3.9–17.2% expression in GC. These CD44v3+ GC cells have CSCs properties as they generate tumorspheres in vitro more efficiently than CD44v3- cells. This is consistent with previous studies showing that CD44v3+/CD24– and CD44v3+/ALDH1+ cells possess CSCs properties in human oral squamous cell carcinoma [18] and HNSCC [19], respectively.

Our transcriptomic analyses showed that two subpopulations of CSCs co-exist in GC, based on their EMT profile as described by Wicha’s group in breast cancer [29], but defined by different CSCs markers: panCD44+ CSCs expressed a proliferative and an epithelial MET-like signature similarly to ALDH+ CSCs in breast cancer [29], and CD44v3+ CSCs harboured an EMT-like signature like CD44+ breast CSCs [29]. In tissues, CD44v3+ GC cells were detected at the invasive front of metastases and in blood vessels as CTCs, whereas panCD44+ GC cells were more centrally located within the tumour mass. ALDH+ CSCs were mainly panCD44+/CD44v3- but a rare population of ALDH+/CD44v3+ cells accounting for ~ 1.3% of cells showed drug efflux-properties highlighting their CSCs chemoresistance properties. Our preliminary data suggest the same results for breast and colon cancer cell lines.

In vivo, both CD44v3+ cells and panCD44+/v3– cells generated heterogeneous tumours consisting of both CD44v3+ and CD44v3– cells, revealing CSCs stemness and plasticity properties. Nevertheless, in some cases panCD44+/v3+ were less tumorigenic in vivo than panCD44+/v3– cells, while it was the contrary in vitro. This discrepancy between in vitro and in vivo results could be attributed to the influence of the tumour microenvironment on CSCs proliferation, plasticity and dormancy properties [34]. Interestingly, it has been reported that the HS site in CD44 v3 exon is involved in metastatic tumour progression but does not affect cell proliferation [13, 14, 35]. Here, we found that CD44v3+ cells remained proliferative compared to CD44v3– cells but were more invasive in vitro and highly metastatic in vivo. Given that Exon v3 was present in combination with all other exons (CD44v2/3-10) and in association with exons v8 to v10 (CD44v3,8-10, Fig.S1), these results are in accordance with the recent findings of Qiu et al. indicating that CD44v3,8-10 are more migrative and invasive in response to the EMT-inducer TGFβ compared to the other CD44v isoforms in GC cell lines [26]. All together, these data suggest that CD44v3-10 and/or CD44v3,8-10 contribute to the metastasis properties of CSC in GC.

In breast and ovarian cancers, physical interactions of CD44v3 with the extracellular matrix component hyaluronic acid and the cytoskeletal protein Ankyrin induce tumour cell migration through the stimulation of Rac1 and RhoA Rho GTPases [31, 36, 37]. Interestingly, CCDC125, that negatively controls Rho GTPases activity and motility [38], was found downregulated in panCD44+ and CD44v3+ cells, suggesting that this pathway could be involved in CD44v3+ cells invasive properties.

We previously demonstrated that carcinogenic strains of *H. pylori* alter the differentiation of gastric epithelial cells, leading to an EMT-like process and emergence of CD44+ cells with CSCs properties [23, 39, 40]. EMT is transient for cell dissemination, and after homing to distant organs, cells need to revert to a more epithelial phenotype to grow, differentiate and develop metastases [41]. EMT is an inducible and highly dynamic process modulated through diverse mechanisms including epigenetic modifications and alternative splicing [42]. Alternative splicing is a key mechanism to increase proteomic diversity and rapidly adapt to microenvironment changes. Recently, CD44v3 expression has been shown to be regulated by epithelial splicing regulatory protein 1 (ESRP1) and involved in pluripotent stem cells maintenance [43]. In our study, *ESRP1* was neither differentially expressed in CD44v3+ cells in transcriptomic analyses nor after CD44v3 silencing by siRNA (Supplemental Fig.S8 A–B). However, mRNA transcripts of *CELF2* RNA-binding protein involved in RNA alternative splicing were significantly upregulated in CD44v3+ cells and downregulated in panCD44+ cells (supplemental results and Fig.S8A). CEFL2 expression was not affected by CD44v3 silencing (Supplemental Fig.S8B), but CELF2 silencing decreased CD44v3 expression independently of ESRP1 whose expression was not affected (Supplemental Fig.S8C). Indeed CELF proteins are mainly considered as tumour suppressors, TCGA data show that CELF2 expression is associated with shorter overall survival in invasive breast carcinoma, low-grade glioma and glioblastoma [44]. Their function in GC has been poorly studied [45] and remains to be investigated. Our data suggest that in GC, CEFL2 may be involved in CD44 alternative splicing for CD44v3 expression. In addition, the analysis of molecular database of GC show that CELF2 but not ESRP1 is associated with shorter overall survival (Supplemental Fig.S8D), maybe at least in part thanks to its role on CD44 alternative splicing and CD44v3 expression.

In patients with colorectal cancer, a subpopulation of CTCs bearing all the functional attributes of CSCs have been described, suggesting that CTCs may derive from CSCs having undergone EMT [32, 33]. CTCs were almost all CD44v6+ and the proportion of CD44v6+/ALDH+ CSCs was higher in CTCs than in primary tumours. These results support a previous study from Todaro et al.having demonstrated that CD44v6 is a marker of CSC-driving colon cancer metastasis [32], probably through its interaction with the c-Met tyrosine kinase receptor. Similarly, CD44v3 splicing variant, via its HS side chain, concentrates HGF and promotes c-Met signalling [46]. Interestingly, we report here that CD44v3+ cells represent a subpopulation among the CD44v6+ cells, suggesting that the metastatic properties of CD44v6+ CSC-driving colon cancer metastasis could be attributed to the subpopulation of CD44v3+ cells among them.

CD44v3 expression has been associated with tumour progression and a poor prognosis in colon cancer [13] and in HNSCC [19], but results from GC patients were controversial [20, 22]. We demonstrate here that CD44v3 protein expression is significantly correlated with distant metastasis, and that GC patients bearing CD44v3+ tumours have a lower 5 years overall survival.

Targeting cell surface markers expressed by CSCs appears as an attractive therapeutic option. Proof of concept of CD44 variants targeting efficiency has emerged from previous studies. Reeder et al. have shown that reducing CD44v3 and CD44v6 expression in colorectal cancer cell lines led to liver metastasis inhibition in xenograft models [35]. However, Bivatuzumab Mertansine, an anti-CD44v6 antibody–drug conjugated, was stopped in phase I clinical trial for HNSCC due to fatal skin toxicity [47]. Interestingly, CD44v3 is less expressed than CD44v6 in several tissues, including the gastrointestinal tract [15], and poorly expressed in the gastric mucosa. Targeting CD44v3 might thus be less toxic than targeting CD44v6 and could constitute an interesting strategy, in addition to conventional treatments, to target invasive CSCs and prevent metastatic dissemination in GC.

In conclusion, CD44v3+ subpopulation of GC cells may correspond to EMT-like CSCs with invasive capacity (Fig. 6D), while panCD44+ GC cells may correspond to more proliferative MET-like CSCs. Further studies are required to assess molecular mechanisms involved in CD44v3 expression as putative targets to prevent metastatic dissemination. Furthermore, detecting CD44v3 expression in primary tumours and maybe blood samples of GC patients would be useful as a prognosis marker and to manage clinical evolution of the disease.

## Supplementary Information

Below is the link to the electronic supplementary material.

Supplementary file1 (DOCX 66 KB)

Supplementary file2 (CSV 1612 KB)

Supplementary file3 (CSV 101 KB)

Supplementary file4 (XLS 54 KB)

Supplementary file5 (CSV 954 KB)

Supplementary file6 (CSV 23 KB)

Supplementary file7 (PDF 2493 KB)

## Data Availability

The datasets generated during and/or analysed durg the current study are available frmo the corresponding author on reasonable request.
