# Supplementary material for: CD44v3 is a marker of invasive cancer stem cells driving metastasis in gastric carcinoma
Source: Gastric Cancer. 2022 Dec 18;26(2):234–49. doi: 10.1007/s10120-022-01357-y (PMC9950191; doi:10.1007/s10120-022-01357-y)
Supplement: Supplementary file 1 — Supplementary file1 (DOCX 66 KB) [file 10120_2022_1357_MOESM1_ESM.docx]

**Supplemental Information**

**SUPPLEMENTARY METHODS**

**Gastric epithelial cell lines culture.** GC cell lines were authenticated by STR profiling and were mycoplasma free (verified by PCR). Cells were cultured in media supplemented with 10% heat-inactivated foetal calf serum and 50 IU/mL of penicillin and 50 mg/L of streptomycin (all from Invitrogen), at 37 °C in a 5% CO2 humidified atmosphere. Media were RPMI 1640-Glutamax for MKN45 and MKN74 and DMEM F12-Glutamax for AGS, KATO III and NCI-N87 ^1^ ^2^ ^3^ ^4^.

**Invasion and gelatine degradation assays.** Adherent cells were recovered by trypsinization and 30.000 48-h siRNA-transfected cells or 5.000 CD44v3+/- FACS-sorted cells were seeded in 8-µm pored Transwell® (Sigma-Aldrich, St. Quentin-Fallavier, France) previously coated with rat-tail type 1 collagen (Becton Dickinson, Le Pont de Claix, France). After 18 h of incubation at 37°C, inserts were ﬁxed with 4% paraformaldehyde (PFA) and processed for DAPI staining. Cells from the upper part of the transwell inserts were removed by swabbing while invaded cells were counted using ZOE^TM^ Fluorescent Cell Imager (Bio-Rad, California, USA). For gelatine degradation assay, 40.000 cells were seeded on glass coverslips previously coated with gelatine from pig skin (Oregon green 488 conjugate, Life Technology)^5^ . Cells were fixed 24 hours later with 4% PFA, actin was stained with phalloidin-Alexa 647 and nuclei were stained with DAPI. Photos were taken using a light microscope and a 40x objective (Nikon, Champigny sur Marne, France) and gelatine degradation areas were measured using ImageJ software.

**Proliferation index determination.** GC cell lines were cultured in adherence conditions and stained with CellTrace^TM^ Violet reagent (Invitrogen) according to standard guidelines. Mitomycin C inhibitor was used as internal control of the experiment. Mean fluorescence intensity (MFI) of CellTrace^TM^ Violet (405-nm excitation and 450/40-nm bandpass emission filter) in CD44v3+ cells and panCD44+ cell stained with APC-labelled antibodies versus their respective negative cells was determined at time of staining (Day 0) and at day 4 post-staining and seeding in adherent plates. Flow cytometry was performed using a BD LSR Fortessa^TM^ instrument and results were analysed using DIVA software (BD). Proliferation index was calculated as following: 2 – (MFI positive or negative cells at day 4 / MFI positive or negative cells at day 0) and data were relativized to negative cells.

**Tissue Microarray conception.**The Tissue Micro Arrays (TMA) were constructed in collaboration with Haut-Lévèque Hospital and the tumor bank of the CHU Bordeaux using formalin-fixed paraffin-embedded tissues (PET) from stomach surgical specimens from 179 patients with GC from 1999 to 2010. The TMA blocks were formed using a Tissue Arrayer MiniCore®3 (Excilone). The areas of interest were selected and cored in triplicate each 1 mm in diameter. Two control tissues were added to each slide, in order to ensure the specificity of the immunostaining: a negative spleen control (no expression of epithelial markers) and a positive duodenum control (expression of epithelial markers and of stem cells markers at the bottom of the crypts). These TMAs include gastric adenocarcinomas of different grade and histological sub-type determined after reanalysis and classification (G. Belleannée, pathologist) according to the last WHO and Lauren’s classification guidelines of gastric tumors, as well as non-tumor gastric mucosa distant from the tumor site (limits of exeresis), which may be healthy or involving different pre-neoplastic lesions (chronic atrophic gastritis, intestinal metaplasia, dysplasia, scored according to the Sydney System criteria). Clinico-biological data including 5-years overall survival were collected.

**Histology and immunohistochemistry staining.** 3 µm-thick tissue sections were prepared from PET blocks ^2^ ^4^. Primary antibodies were incubated for 0.5 to 2 h at room temperature (RT), followed by 30 min incubation at RT with anti-mouse and anti-rabbit Labelled Polymer-HRP DAKO Envision Systems (DAKO). Immunolabelling was revealed by 1 to 10 min incubation in liquid substrate-diaminobenzidine-chromogen (DAKO). Slides were counterstained with haematoxylin, dehydrated and mounted with Eukitt-mounting medium.

**Microarray gene expression studies.** FACS-sorted cells were recovered in simplicates (n=4 PDX cases and n=1 GC cell line) and in three independent replicates (n=2 PDX cases). RNAs were extracted using RNeasy microkit (All from Qiagen) containing a DNA digestion step. RINs were determined on the TapeStation (Agilent). Gene expression profiles were performed at the GeT‐TRiX facility (GénoToul, Génopole Toulouse Midi-Pyrénées) using Agilent Sureprint G3 Human microarrays (8x60K, design 072363) following the manufacturer's instructions. For each sample, Cyanine-3 (Cy3) labelled complementary RNA (cRNA) was prepared from 25 ng of total RNA using the One-Color Quick Amp Labelling kit (Agilent) according to the manufacturer's instructions, followed by Agencourt RNAClean XP (Agencourt Bioscience Corporation, Beverly, Massachusetts). Dye incorporation and cRNA yield were checked using Dropsense™ 96 UV/VIS droplet reader (Trinean, Belgium). 600 ng of Cy3-labelled cRNA were hybridized on the microarray slides following the manufacturer’s instructions. Immediately after washing, the slides were scanned on Agilent G2505C Microarray Scanner using Agilent Scan Control A.8.5.1 software and fluorescence signal extracted using Agilent Feature Extraction software v10.10.1.1 with default parameters.

**Microarray data statistical analysis.** Microarray data were analysed using R (R Development Core Team, 2008) and Bioconductor packages (www.bioconductor.org, v 3.0) ^6^. Raw data (median signal intensity) were filtered, log2 transformed, corrected for batch effects (microarray washing bath serials) and normalized using quantile method^7^. A model was fitted using the limma lmFit function^8^. For panCD44 negative *vs* positive contrast specific to GC04 or GC10 patients, we fitted 2 models including the “source_tri” as a blocking factor. To investigate common signatures across multiple sources (patient, cell line) the expression data from 3 replicated “tri” in each of GC04 and GC10 patient were averaged. Then 3 models were fitted considering “source” as blocking factor to extract respectively panCD44 negative versus positive, CD44v3 negative *vs* positive signatures. A correction for multiple testing was applied using Benjamini-Hochberg procedure ^9^ for False Discovery Rate (FDR). Probes with FDR ≤ 0.05 were considered to be differentially expressed between conditions. Hierarchical clustering was applied to the samples and the differentially expressed probes using 1-Pearson correlation coefficient as distance and Ward’s criterion for agglomeration. The clustering results are illustrated as a heatmap of expression signals for which the maximum distance method was used for the clustering of individuals.

**Orthotopic tumour model and bioluminescence imaging**. Detailed surgery procedures and bioluminescence imaging were previously described^10^. Briefly, mice received 300 μg/kg buprenorphine (Vetergesic, Centavet) 30 min before laparotomy. Animals were anesthetized with isoflurane in sterile condition, incision was made on the right side of the mouse and 30 μl of medium containing cells were injected into the sub-serosa. Mice were monitored up twice a week by *in vivo* imaging (Biospace Lab, France) upon intraperitoneal injection of 3.3 mg D-Luciferin (Promega, Charbonnières-les-Bains, France). At end point, mice were sacrificed, and organs were macroscopically analysed and collected for histological analyses. In MKN45 expressing luciferase, photon emission on organs was measured after 5 minutes incubation in a solution of 3 mg/mL D-luciferin.

**RNA extraction and RT-qPCR.** RNAs were extracted using Trizol reagent (Ambion). Reverse transcription was performed with 0.5 µg total RNA using the Quantitect Reverse Transcription kit (Qiagen). Quantitative PCR was performed using the SYBR-qPCR-Premix Ex-Taq (TAKARA), 0.3 µmol/L of specific primers and 1:100 of RT reaction volume. Relative expressions were calculated using the comparative Ct method with both *HRPT1* and *TBP* as normalizers. Human qPCR primer sequences are in Supplementary Table 1.

**CD44 exon specific PCR.** Reverse transcription was performed with primer R2 specific to CD44 gene (5’-ATGCAAACTGCAAGAATC-3’,). Primers are listed in Supplementary Table S2. Amplification cycle was the following: Activation: 2’ at 95°C; Amplification (35 cycles): 30” at 95°C - 2’45” at 56°C - 1’ at 73°C; Final extension: 10’ at 73°C.

**KMplot database analysis.**

The following JetSet best probes, 227178_at (CELF2) and 225846_at (ESRP1) were used. P-values were calculated by a log rank test^11^.

**Statistical analysis.** Quantification values represent the mean of three or more independent experiments, each performed in duplicate or more ± S.E.M, as indicated in the legends of the figures. Mann-Whitney test was used to compare two groups of data and Kruskal-Wallis test with Dunn’s post-test or ANOVA test with Bonferroni post-hoc test was used for multiple comparison tests. Association of CD44v3 expression with healthy/pre-cancerous/adenocarcinoma lesions and with the TNM stage of GC patients was assessed using Pearson’s Chi square test. Five–year survival difference between subgroups of CD44v3- and CD44v3+ tumours was determined in Kaplan-Meier survival method by the log rank test. Statistics were performed on GraphPad Prism 6 (USA). * correspond to two-sided *P* value < 0.05.

**SUPPLEMENTARY RESULTS**

Enrichment in mRNA corresponding to panCD44 and CD44v3-containing isoforms was verified in FACS-sorted cells by qRT-PCR (Supplementary Fig.S3C-D). mRNA transcripts of 2,845 probes were increased in panCD44+ cells and 310 in CD44v3+ cells compared to their respective negative counterparts (Fig.3A and Supplementary Tables S4 and S5). Among them, only CD44 and KPNA2 genes were in common (Supplementary Table 6). Similarly, transcripts of 2,578 and 34 probes were downregulated in panCD44+ and CD44v3+ cells, respectively, compared to their respective negative cells (Supplementary Tables S4 and S5), among which, only 6 were in common (Fig.3B and Supplementary Table S6). Enriched signalling pathways of the panCD44+ profile included ribosome biogenesis, DNA replication, RNA polymerase, genes involved in mismatch and nucleotide excision repair and metabolic pathways (pyruvate, pyrimidine and purine) and cell cycle (KEGG analysis, Supplementary Table S7). Interestingly, a significant upregulation of SLC7A11 encoding xCT, a glutamate-cysteine transporter that interacts with CD44E and controls the intracellular level of reduced glutathione and prevents reactive oxygen species-related damage^12^, was observed, which might contribute to the chemoresistance properties of these cells that we previously reported^1^. Enriched signalling pathways of CD44v3+ profile was largely different and included pathways associated with auto-immunity, innate immunity and cell adhesion molecules (Supplementary Table S8).

Among the 310 mRNA transcripts that were significantly upregulated in CD44v3+, 60 were significantly downregulated in panCD44+ (Fig.3A-B, Supplementary Table S6). Interestingly, many of them have been involved in EMT, invasion and tumour aggressiveness, including Zinc finger E-box-binding homeobox 2 (*ZEB2*) ^13^, Rac Family Small GTPase 2 (*RAC2*)^14^, Transforming Growth Factor alpha (*TGFA*), Neurogenin 3 (*NEUROG3*), Tetraspanin 10 (*TSPAN10*) ^15^, Myristoylated Alanine-Rich protein Kinase C Substrate (*MARKS*)^16–18^, ST8 Alpha-N-Acetyl-Neuraminide Alpha-2,8-Sialyltransferase 1 (*ST8SIA1*)^19^, the brain abundant, membrane attached signal protein 1 (*BASP1*)^20^, Histone Cluster 1 H2a family (*HIST1H2A*)^21^ and variants^22^, while others are RNA binding proteins involved in post-transcriptional modifications including pre-mRNA alternative splicing, ie, CUG-BP Elav-Like Family Members -2 (*CELF2*) ^23–25^.

Altogether, these results suggest that panCD44+ and CD44v3+ subpopulations of GC cells express distinct patterns of genes, panCD44+ cells harbouring a more metabolic signature while CD44v3+ cells harboured a signature more related to EMT.

**SUPPLEMENTARY FIGURES**

**Figure S1.** Detection of a panel of CD44 isoforms in GC compared to normal gastric mucosa. (**A**) Schematic representation of human *CD44* pre-messenger from exon 5 to exon 16. Orange boxes represent constitutive exons included in CD44 mRNA and blue boxes represent variable exons that are submitted to alternative splicing. Arrows represent primers used to performed exon-specific PCR (blue arrows indicate primers located in variable exons in forward position; orange arrows indicate primers located in constant exons in reverse position). (**B**) RT-PCR exon specific analysis of *CD44* variants (v2, v3a, v3b, v4, v5, v6, v7, v8, v9, v10) and total isoforms (first line) in GC cell lines (MKN45 and NCI-N87) and PDX cells (GC04, GC06, GC10). RT-PCR of RNA 18S was performed on GC cell lines and PDX cDNA or water (RTO) as internal controls. (**C**) Relative mRNA expression measured by qRT-PCR of diverse CD44 isoforms (CD44S, CD44E, CD44-containing exon v3 including CD44v3, CD44v3E) and total isoforms of CD44 (CD44t) in normal mucosa from sleeve gastrectomies (normal fundus and antrum parts of the stomach, purple bars), in GC cell lines (green bars) and in PDX cells (orange bars). *HPRT1* and *TBP* quantification were used as endogenous controls. §, expression not detected.

**Figure S2**. Expression of CD44 isoforms is enriched in chemo-resistant cells. (**A-B**) Relative mRNA level of diverse CD44 isoforms (CD44S, CD44E, CD44v3) and total CD44 (CD44t) measured by qRT-PCR in chemo-resistant GC cell lines (**A**) and PDX cells (**B**). Cells were treated (50 µmol/L 5-Fluorouracil, 5-FU, grey bars; 1 µmol/L doxorubicin, white bars) or not (Control, black bars) during 48 h for cells cultured in adherent condition (**A**) and during 72 h for cells cultured in tumorsphere condition (**B**). Data are mean ± SEM of ≥ 3 independent experiments.

**Figure S3**. Differentially expressed genes according to the expression of panCD44 and CD44v3 in GC samples. (**A**) Hierarchical clustering of 7 samples and 5,423 genes differentially expressed (adjusted *p* values *P<0.05*) between panCD44+ cells and panCD44- cells isolated by FACS. Each row represents a gene and each column represents a sample (value for GC04 and GC10 correspond to the mean of each triplicates). (**B**) Hierarchical clustering of 5 samples and 344 genes differentially expressed (adjusted *p* values *<0.05*) between CD44v3+ cells and CD44v3- cells isolated by FACS. Each row represents a gene and each column represents a sample. (**C-D**) Validation of samples used for transcriptome analysis by analysing mRNA expression of total CD44 (**C**) and CD44v3 (**D**) by qRT-PCR in cells sorting by FACS (panCD44+ cells versus panCD44- cells and CD44v3+ cells versus CD44v3- cells, respectively).

**Figure S4. (A)** Correlation of the percentage of panCD44+ cells with the areas (x10^4^ µm^2^) of the lung and liver metastases developed 8 weeks after orthotopic injection of GC10 cell in NSG mice (n=4 mice with a total of 152 micro/macro metastases in the lung and 31 in the liver were analysed). (**B**) Normalized cell number to the day before treatment (D0) and to control untreated condition of CD44v3^-^ and CD44v3^+^ FACS-sorted cells upon 96 h 5-fluorouracile (5-FU) and doxorubicin (DOXO)-treatment. Data are mean ± SD of 5 replicates.

**Figure S5.** Validation of 2 siRNA down-regulating CD44v3 expression. (**A**) Relative mRNA level of CD44 isoforms (CD44S, CD44E, CD44v3, CD44v3E) and total CD44 (CD44t) measured by qRT-PCR in MKN45 cells (upper panel) and NCI-N87 cells (lower panel) transfected with siCD44v3 (siCD44v3-1 and siCD44v3-2) and compared with control siRNA (siCtrl). Data are mean ± S.E.M of ≥ 3 independent experiments. (**B**) Representative immunofluorescence images of CD44v3 (green), actin stained with phalloidin (white) and nuclei stained with DAPI (blue) in MKN45 and NCI-N87 cell transfected with siCtrl and siCD44v3-1. Scale bars, 10 µM. (**C**) Percentage of invasive GC07 cells counted 24 h after the second round of siRNA transfection (control siRNA compared to siCD44v3-1 and siCD44v3-2) and seeded for 18 hours in collagen coated Transwells®. Data are mean ± S.E.M of 3 independent experiments realized in triplicates. (**D**) Percentage of degradation area of MKN45 and NCI-N87 cells measured 24 h after the second round of siRNA transfection (control siRNA compared to siCD44v3-1 and siCD44v3-2) and seeded for 24 h on gelatin-coated coverslips. Data are mean ± S.E.M of 3 independent experiments realized in duplicates.

**Figure S6.** CD44v3+ cells represent a subpopulation of CD44V6+/V9+ cells. (**A**) Flow cytometry profiles for CD44v6 and CD44v9 on CD44v3+ and CD44v3- cells. (**B-D**) Flow cytometry profiles for CD44v3 and CD44v6 on human GC cell lines (**B**), colon cancer cell lines (**C**) and breast cancer cell lines (**D**). (**E**) Representative images of CD44v3 (upper panels) and CD44v6 (lower panels) detected by immunohistochemistry on human gastric PDX grown subcutaneously in NSG mice. Scale bars, 50 µm.

**Figure S7.** (**A-B**) Representative images of CD44v3 and CD44v6 isoforms detection by immunohistochemistry performed on human colon tumours, lymph node and liver metastases (**A**) and on breast tumours and lymph node metastases (**B**). (**C, E**) Correlation between CD44v3 and CD44v6 expression in human primary colon tumours (n=7, left panel) and distant metastases (liver and lymph node, n=7 each, right panel) (**C**) and in human primary breast tumours (n=11, left panel) and distant lymph nodes metastases (n=11, right panel) (**E**). R, Pearson correlation coefficient. (**D**, **F**) Expression scores of CD44v3 and CD44v6 in primary colon tumours, lymph node and liver metastases (n=7 patients, **D**) and in primary breast tumours and lymph node metastases (n=11 patients, **F**). Data are mean ± S.E.M.

**Figure S8.** (**A**) Heatmap of ESPR1 and CELF2 expression as described in figure 3C. (**B-C**) Relative mRNA level of ESPR1, CELF2, CD44v3, CD44v3E and CD44S measured by qRT-PCR in MKN45 cells transfected with siCD44v3 (B) or siCELF2 (C) and compared with control siRNA (siCtrl). Data are mean ± S.E.M of 4 independent experiments in B and 2 independent experiments in C, each performed at least in triplicate. (**D**) Kaplan-Meier survival plots association with the expression of CELF2 and ESPR1 in GC patients (n=631).

**SUPPLEMENTARY TABLES**

**Supplementary Table S1. List of primers used for qRT-PCR analysis.**

| **Gene** | **Sequence Frame (5'-3')** | **Sequence Reverse (5'-3')** |
| --- | --- | --- |
| ***ALDH1A1*** | CAAGATGTCTGGAAATGGAAGAGA | TGACTGTTTTGACCTCTGTATATTCATG |
| ***CLDN3*** | GAGGGCCTGTGGATGAACTG | AGCGAGTCGTACACCTTGCA |
| ***CD24*** | TTCTCCAAGCACCCAGCA | TGGAATAAATCTGCGTGGGTA |
| ***CD44*** | CGGACACCATGGACAAGTTT | GAAAGCCTTGCAGAGGTCAG |
| ***CD44E*** | AGAATCCCTGCTACCAATATGGACTC | AGGTCACTGGGATGAAGGTC |
| ***CD44S*** | CATCTACCCCAGCAACCCTA | CTTGGTCTCTGGTAGCAGGGA |
| ***CD44v3*** | GCACTTCAGGAGGTTACATC | CTGAGGTGTCTGTCTCTTTC |
| ***CD44v3E*** | TATCTCCAGCACCAATATGG | CATCATTCCTATTGCTTGATGTC |
| ***CELF2*** | CTGATTCTTCTGTCCTCATTGTGAA | CTGCATCACACAATAACATACATTACAAA |
| ***CTNNB1*** | CAGCTGCTGTTTTGTTCCGAA | CAGCTCAACTGAAAGCCGTTT |
| ***DSP*** | CACCAGCCCCCCTCTCA | AAGGAGACTCATTCCAAAATGCTT |
| ***ESRP1*** | CAGAGGCACAAACATCACAT | AGAAACTGGGCTACCTCATTGG |
| ***HPRT1*** | TGGTCAGGCAGTATAATCCA | GGTCCTTTTCACCAGCAAGCT |
| ***SNAI1*** | ACAATGTCTGAAAAGGGACTGTGA | CAGACCAGAGCACCCCATT |
| ***TBP*** | GGGCATTATTTGTGCACTGAGA | GCCCAGATAGCAGCACGGT |
| ***TGFB1*** | CCTGGACACCAACTATTGCTTCA | CCTTGCGGAAGTCAATGTACAG |
| ***VIMENTIN*** | GGATGCCCTTAAAGGAACCAA | CAACGGCAAAGTTCTCTTCCAT |
| ***ZEB1*** | TCCCAACTTATGCCAGGCAC | CAGGAACCACATTTGTCATAGTCAC |
| ***ZEB2*** | TGCAAGTGCCATCCTTGTACA | CAAAGAACAGGGTGAGCTTAACAC |

**Supplementary Table S2. List of primers used for RT-PCR exon specific analysis.**

| **Probe name** | **Sequence Frame (5'-3')** |
| --- | --- |
| **Exon 16-R1** | TTTGCTCCACCTTCTTGACTCC |
| **Exon 5-F1** | AAGACATCTACCCCAGCAAC |
| **Exon v2-F** | GATGAGCACTAGTGCTACAG |
| **Exon v3a-F** | ACGTCTTCAAATACCATCTC |
| **Exon v3b-F** | TGGGAGCCAAATGAAGAAAA |
| **Exon v4-F** | TCAACCACACCACGGGCTTT |
| **Exon v5-F** | GTAGACAGAAATGGCACCAC |
| **Exon v6-F** | CAGGCAACTCCTAGTAGTAC |
| **Exon v7-F** | CAGCCTCAGCTCATACCAGC |
| **Exon v8-F** | TCCAGTCATAGTATAACGCT |
| **Exon v9-F** | CAGAGCTTCTCTACATCACA |
| **Exon v10-F** | GGTGGAAGAAGAGACCCAAA |

**Supplementary Table S3. List of siRNA sequences.**

| **siRNA name** | **Sequence Frame (5'-3')** |
| --- | --- |
| **Non-silencing control (siCtrl)** | GGGCAAGACGAGCGGGAAG |
| **human siCD44v3-1** | AAGAGACAGACACCUCAGU |
| **human siCD44v3-2** | AAUACCAUCUCAGCAGGCU |
| **human siCELF2-1** | ACCCGCAUCAUUACAGUCCAA |
|  | CACGUUUAGUGCUAUGUCCUA |
| **human siCELF2-2** | AUCCACGCAGUUCUUAACAUA |
|  | CAGGCCUACUCAGGAAUUCAA |

**SUPPLEMENTARY REFERENCES**

1. Nguyen PH, Giraud J, Chambonnier L, et al. Characterization of Biomarkers of Tumorigenic and Chemoresistant Cancer Stem Cells in Human Gastric Carcinoma. Clin Cancer Res Off J Am Assoc Cancer Res 2017;23:1586–1597.

2. Nguyen PH, Giraud J, Staedel C, et al. All-trans retinoic acid targets gastric cancer stem cells and inhibits patient-derived gastric carcinoma tumor growth. Oncogene 2016;35:5619–5628.

3. Tiffon C, Giraud J, Molina-Castro SE, et al. TAZ Controls Helicobacter pylori-Induced Epithelial-Mesenchymal Transition and Cancer Stem Cell-Like Invasive and Tumorigenic Properties. Cells 2020;9:E1462.

4. Molina-Castro SE, Tiffon C, Giraud J, et al. The Hippo Kinase LATS2 Controls Helicobacter pylori-Induced Epithelial-Mesenchymal Transition and Intestinal Metaplasia in Gastric Mucosa. Cell Mol Gastroenterol Hepatol 2020;9:257–276.

5. Varon C, Tatin F, Moreau V, et al. Transforming growth factor beta induces rosettes of podosomes in primary aortic endothelial cells. Mol Cell Biol 2006;26:3582–3594.

6. Gentleman RC, Carey VJ, Bates DM, et al. Bioconductor: open software development for computational biology and bioinformatics. Genome Biol 2004;5:R80.

7. Bolstad BM, Irizarry RA, Astrand M, et al. A comparison of normalization methods for high density oligonucleotide array data based on variance and bias. Bioinforma Oxf Engl 2003;19:185–193.

8. Smyth GK. Linear models and empirical bayes methods for assessing differential expression in microarray experiments. Stat Appl Genet Mol Biol 2004;3:Article3.

9. Benjamini Y, Drai D, Elmer G, et al. Controlling the false discovery rate in behavior genetics research. Behav Brain Res 2001;125:279–284.

10. Giraud J, Bouriez D, Seeneevassen L, et al. Orthotopic Patient-Derived Xenografts of Gastric Cancer to Decipher Drugs Effects on Cancer Stem Cells and Metastatic Dissemination. Cancers 2019;11.

11. Szász AM, Lánczky A, Nagy Á, et al. Cross-validation of survival associated biomarkers in gastric cancer using transcriptomic data of 1,065 patients. Oncotarget 2016;7:49322–49333. Available at: https://www.oncotarget.com/article/10337/ [Accessed November 17, 2022].

12. Ishimoto T, Nagano O, Yae T, et al. CD44 variant regulates redox status in cancer cells by stabilizing the xCT subunit of system xc(-) and thereby promotes tumor growth. Cancer Cell 2011;19:387–400.

13. Fardi M, Alivand M, Baradaran B, et al. The crucial role of ZEB2: From development to epithelial-to-mesenchymal transition and cancer complexity. J Cell Physiol 2019.

14. Liu Y, Cheng G, Song Z, et al. RAC2 acts as a prognostic biomarker and promotes the progression of clear cell renal cell carcinoma. Int J Oncol 2019;55:645–656.

15. Haining EJ, Yang J, Bailey RL, et al. The TspanC8 subgroup of tetraspanins interacts with A disintegrin and metalloprotease 10 (ADAM10) and regulates its maturation and cell surface expression. J Biol Chem 2012;287:39753–39765.

16. Mohapatra P, Yadav V, Toftdahl M, et al. WNT5A-Induced Activation of the Protein Kinase C Substrate MARCKS Is Required for Melanoma Cell Invasion. Cancers 2020;12:E346.

17. Chen C-H, Thai P, Yoneda K, et al. A peptide that inhibits function of Myristoylated Alanine-Rich C Kinase Substrate (MARCKS) reduces lung cancer metastasis. Oncogene 2014;33:3696–3706.

18. Quan R, Ning Z, Wang Y, et al. Prognostic Value of Upregulation of Myristoylated Alanine-Rich C-Kinase Substrate in Gastric Cancer. Med Sci Monit Int Med J Exp Clin Res 2019;25:279–287.

19. Nguyen K, Yan Y, Yuan B, et al. ST8SIA1 Regulates Tumor Growth and Metastasis in TNBC by Activating the FAK-AKT-mTOR Signaling Pathway. Mol Cancer Ther 2018;17:2689–2701.

20. Tang H, Wang Y, Zhang B, et al. High brain acid soluble protein 1(BASP1) is a poor prognostic factor for cervical cancer and promotes tumor growth. Cancer Cell Int 2017;17:97.

21. Liu S, Cong Y, Wang D, et al. Breast Cancer Stem Cells Transition between Epithelial and Mesenchymal States Reflective of their Normal Counterparts. Stem Cell Rep 2014;2:78–91. Available at: https://www.sciencedirect.com/science/article/pii/S2213671113001495 [Accessed March 11, 2021].

22. Lone IN, Sengez B, Hamiche A, et al. The Role of Histone Variants in the Epithelial-To-Mesenchymal Transition. Cells 2020;9:E2499.

23. Piqué L, Martinez de Paz A, Piñeyro D, et al. Epigenetic inactivation of the splicing RNA-binding protein CELF2 in human breast cancer. Oncogene 2019;38:7106–7112.

24. Yeung YT, Fan S, Lu B, et al. CELF2 suppresses non-small cell lung carcinoma growth by inhibiting the PREX2-PTEN interaction. Carcinogenesis 2020;41:377–389.

25. Dasgupta T, Ladd AN. The importance of CELF control: molecular and biological roles of the CUG-BP, Elav-like family of RNA-binding proteins. Wiley Interdiscip Rev RNA 2012;3:104–121.
