# Supplementary figures and images for: CD44v3 is a marker of invasive cancer stem cells driving metastasis in gastric carcinoma
Source: Gastric Cancer. 2022 Dec 18;26(2):234–49. doi: 10.1007/s10120-022-01357-y (PMC9950191; doi:10.1007/s10120-022-01357-y)

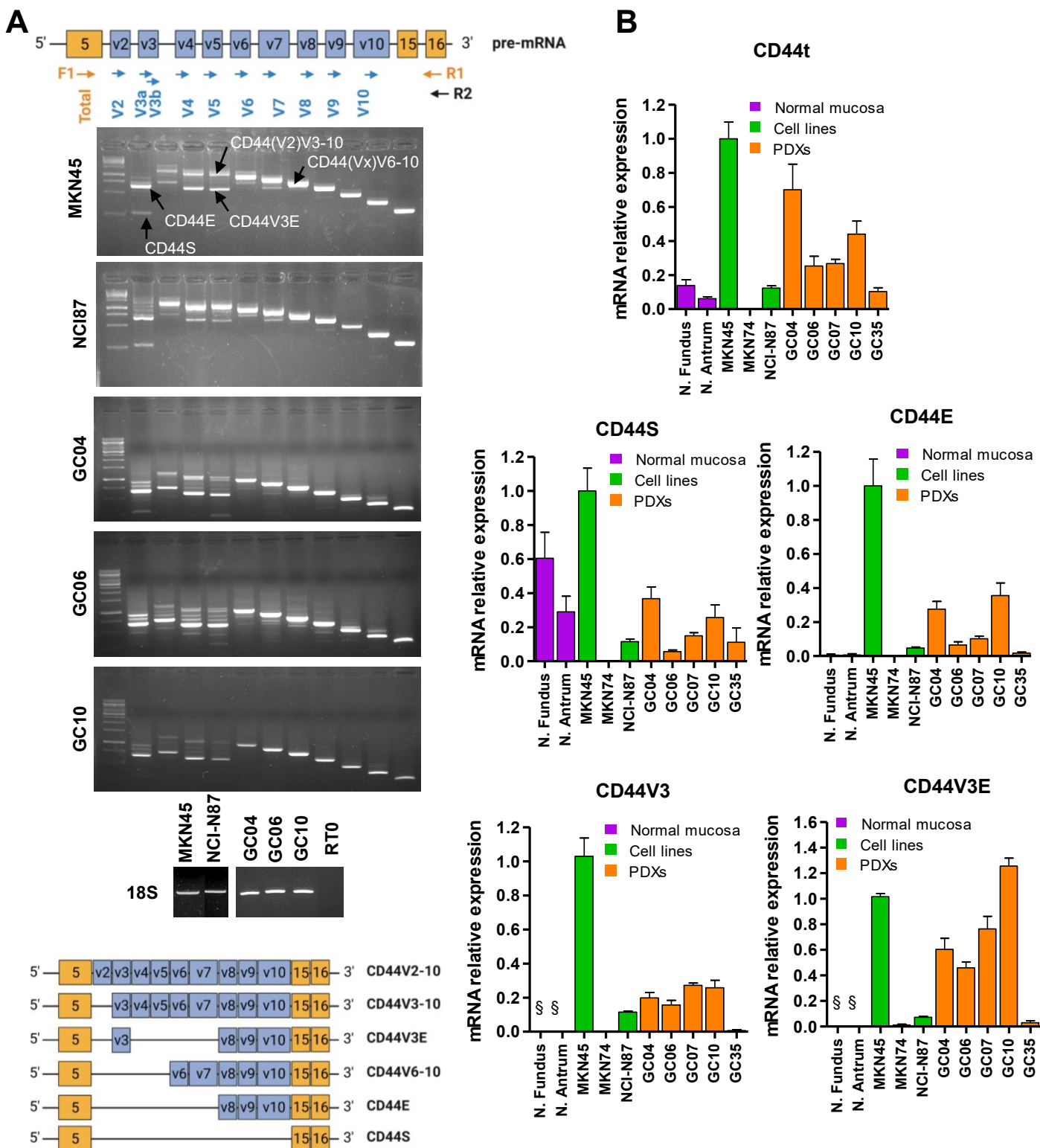

Figure S1

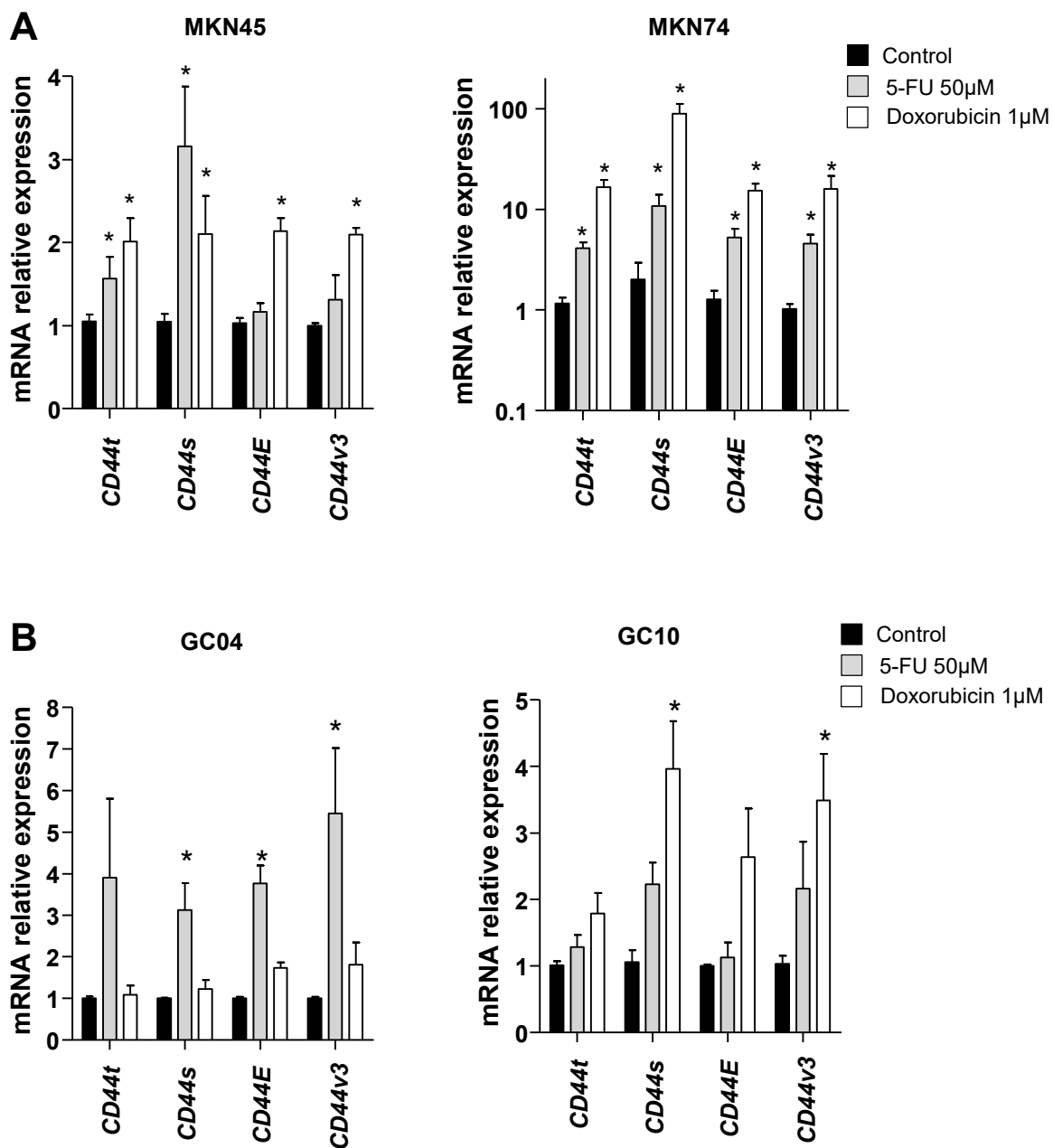

**Figure S2**

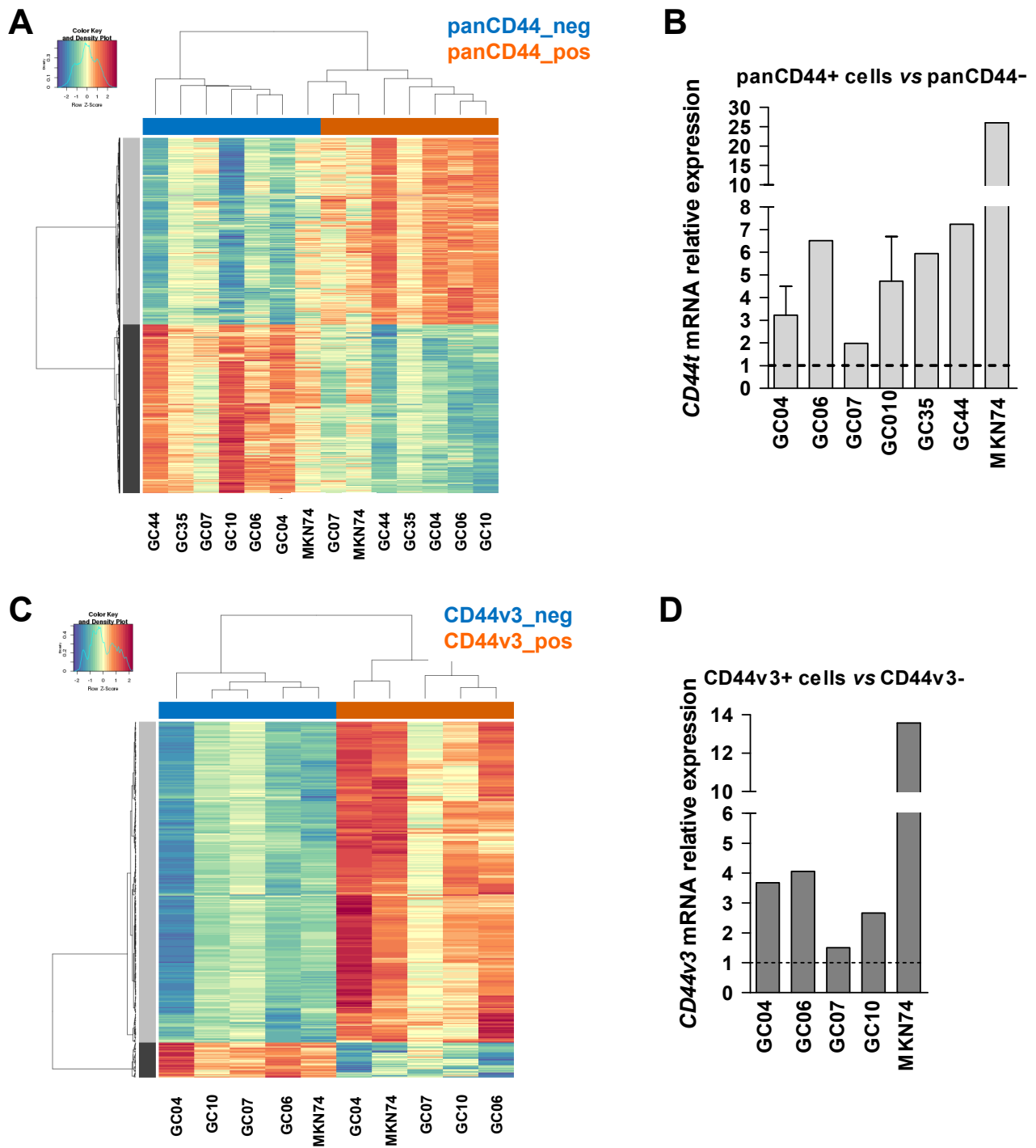

**Figure S3**

**A**

**Case GC10**

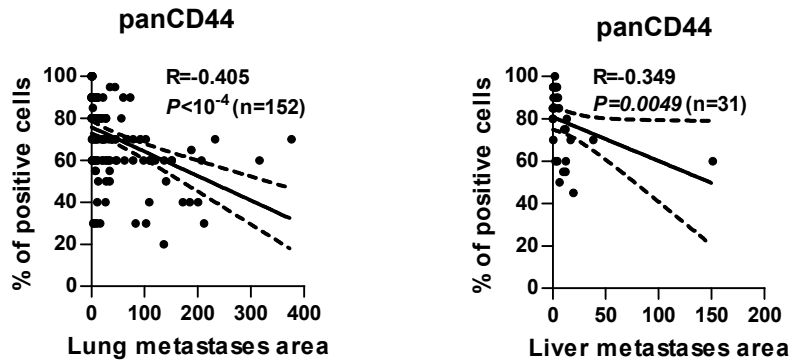

**B**

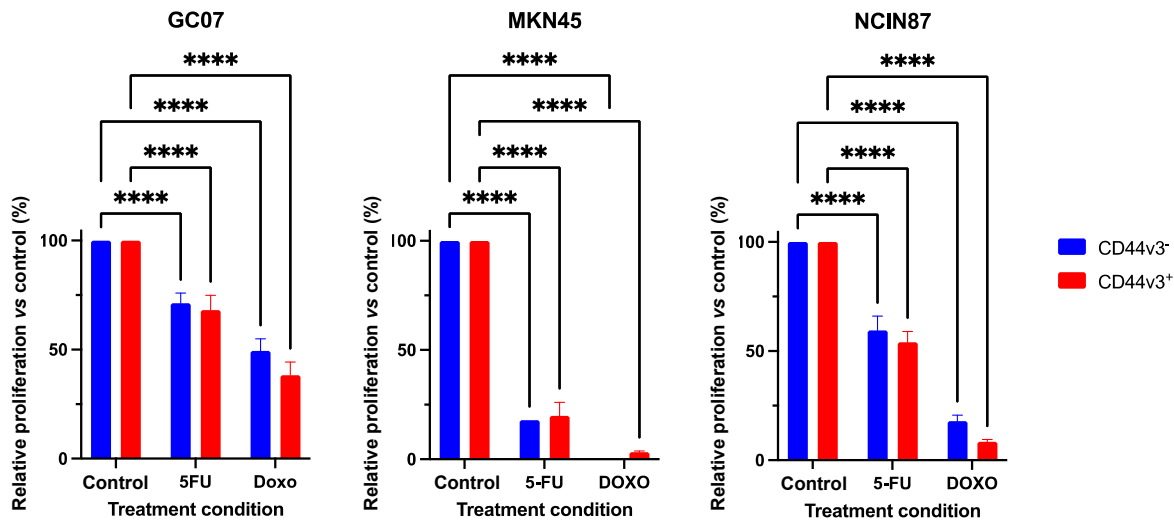

**Figure S4**

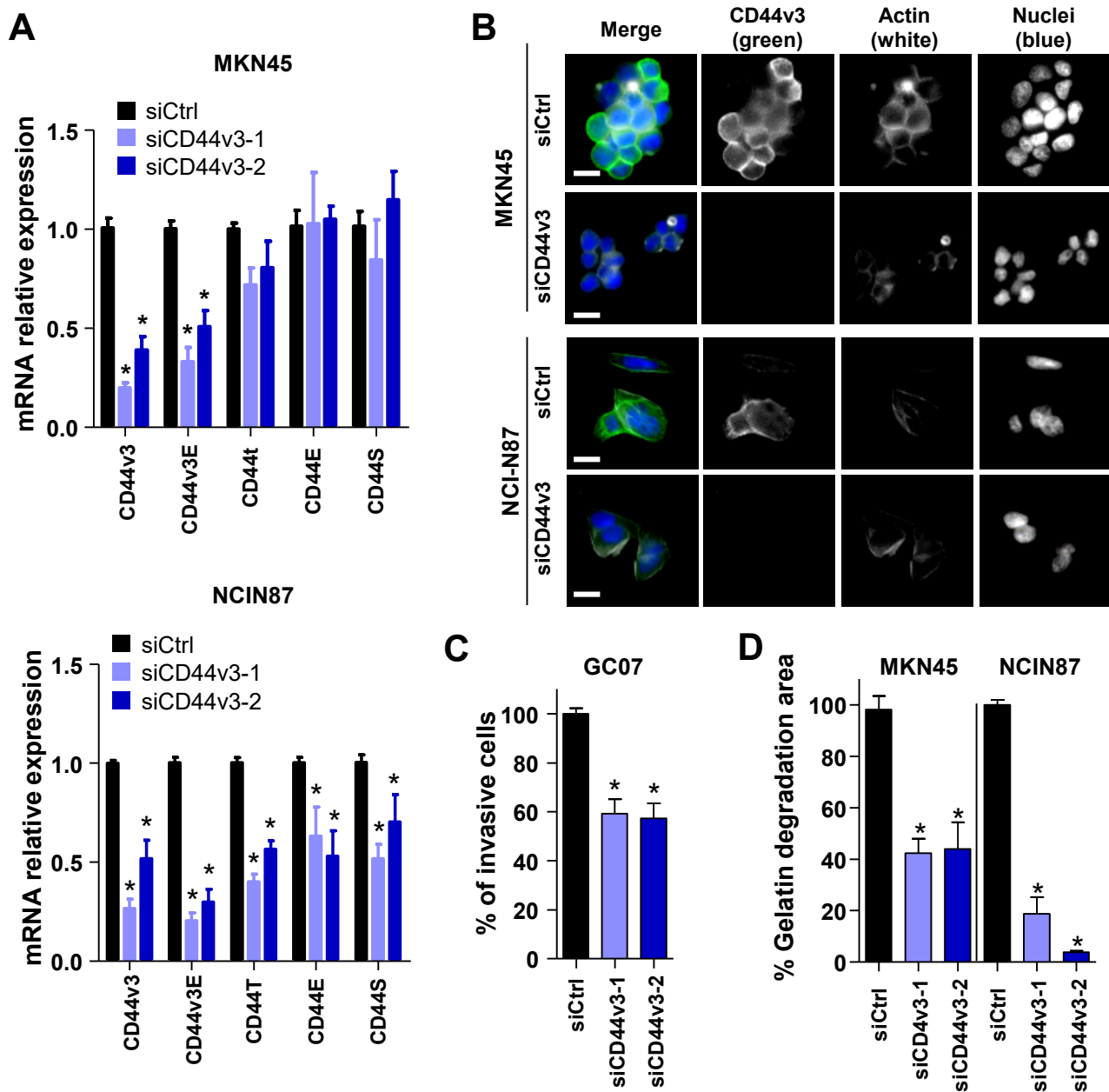

**Figure S5**

**A**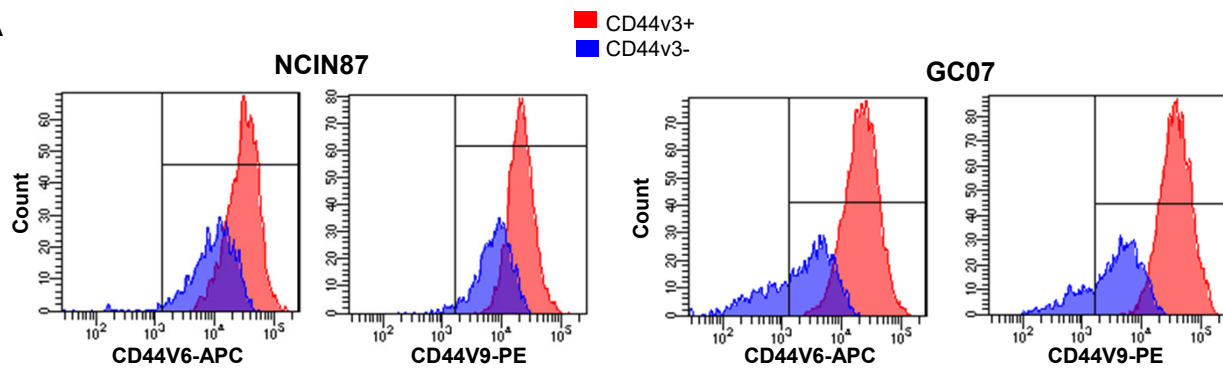**B**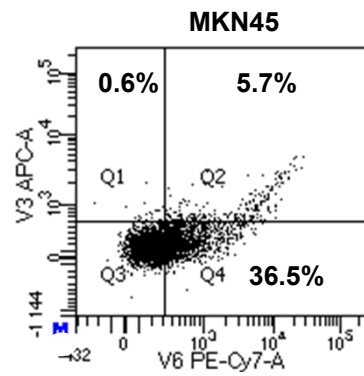**C**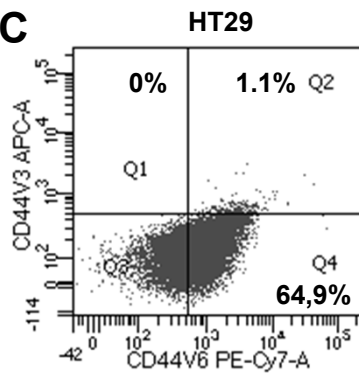**D**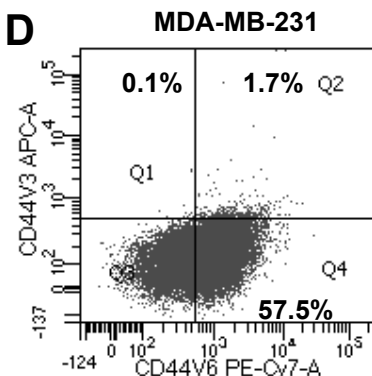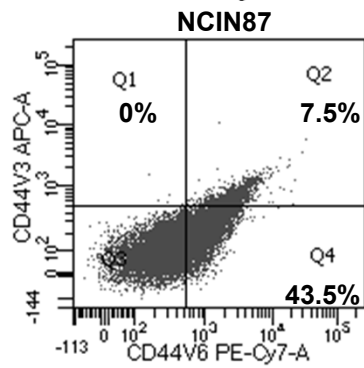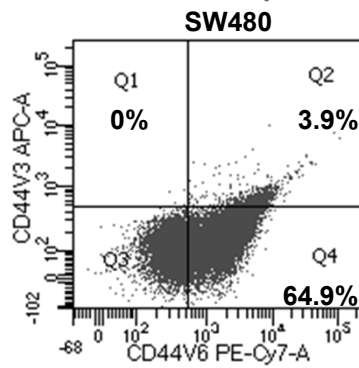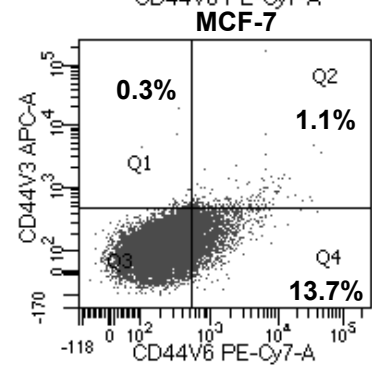**E**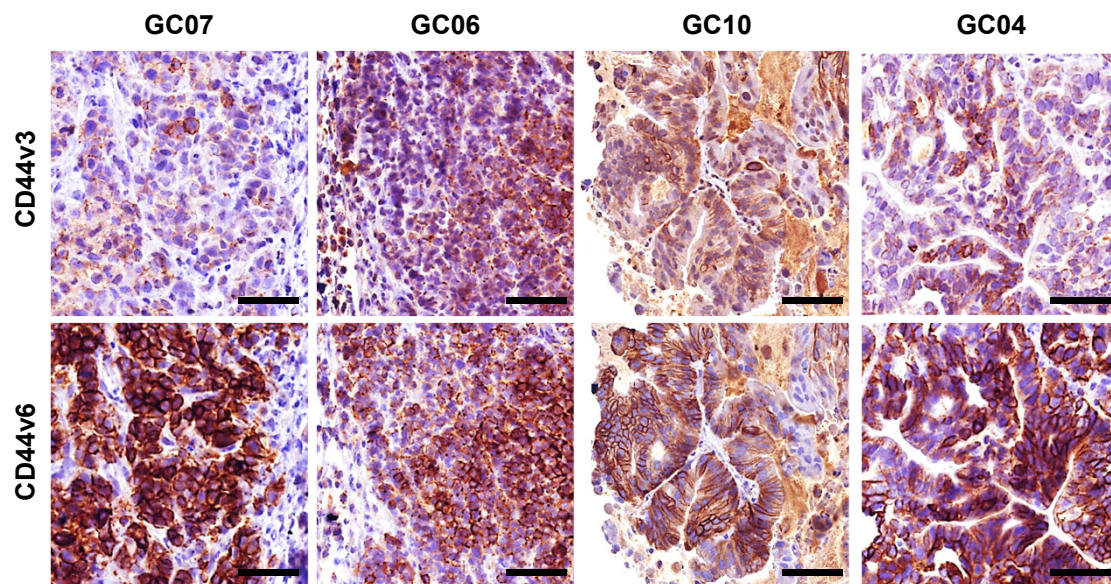**Figure S6**

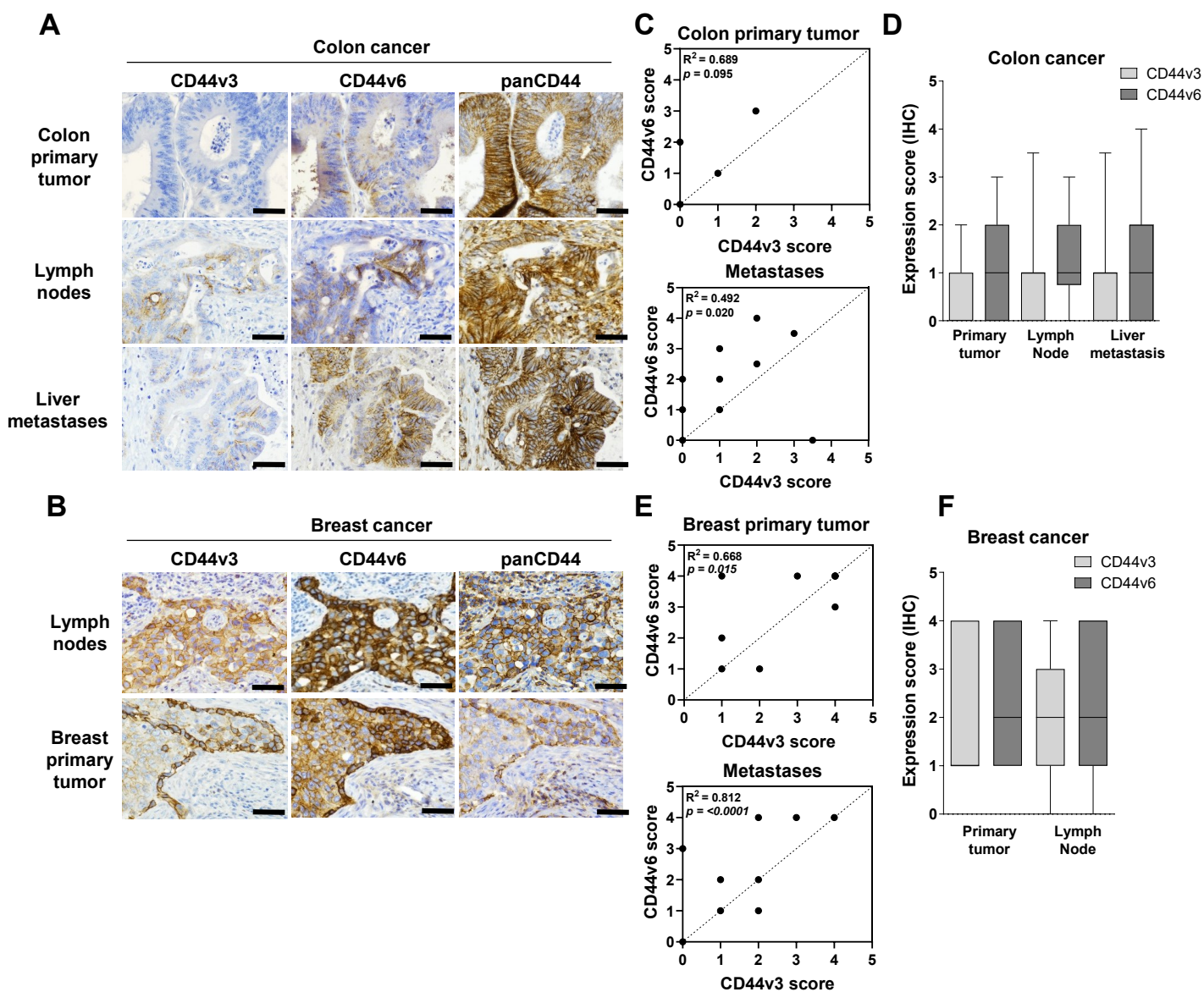

**Figure S7**

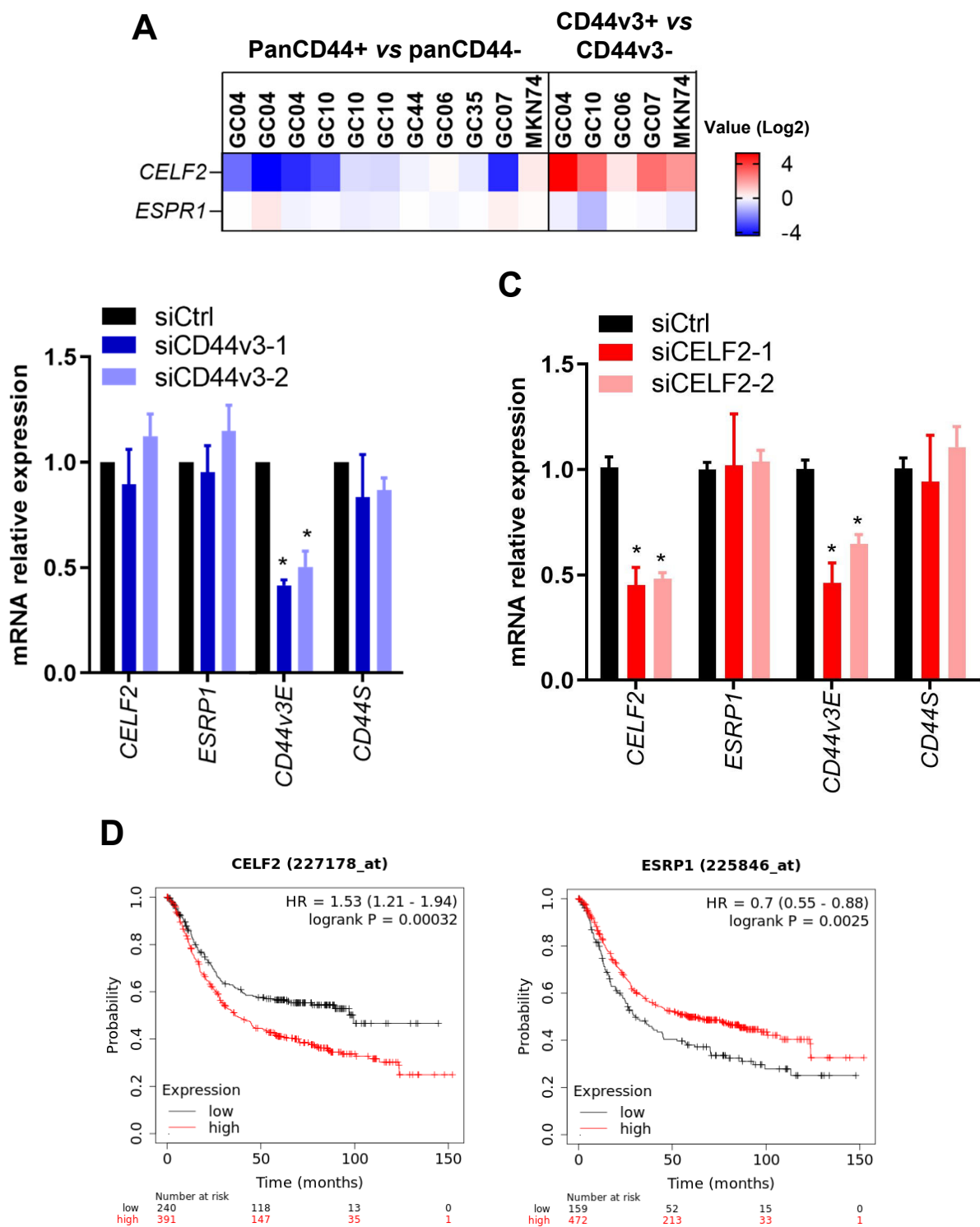

**Figure S8**

Supplement: Supplementary file 7 — Supplementary file7 (PDF 2493 KB) [file 10120_2022_1357_MOESM7_ESM.pdf]
